# Spatial transcriptome and single-cell reveal the role of nucleotide metabolism in colorectal cancer progression and tumor microenvironment

**DOI:** 10.1186/s12967-024-05495-y

**Published:** 2024-07-29

**Authors:** Junzhi Liu, Huimin Li, Lantian Wang, Shurui Wang, Qiang Tang

**Affiliations:** 1 The Second Affiliated Hospital of Zhejiang University School Medicine, Hang Zhou, 310000 China; 2 Chinese Academy of Medical Sciences & Peking Union Medical College, Beijing, 100730 China

**Keywords:** Single cell RNA-sequencing, Spatial transcriptomics, Colorectal cancer, Nucleotide metabolism, Fibroblasts, NME1

## Abstract

**Background:**

The intricacies of nucleotide metabolism within tumor cells specific to colorectal cancer (CRC) remain insufficiently characterized. A nuanced examination of particular tumor clusters and their dynamic interplay with the tumor microenvironment (TME) may yield profound insights into these therapeutically auspicious communicative networks.

**Methods:**

By integrating ten types of single-cell enrichment scoring methods, we carried out enrichment analysis on CRC cell types, which was validated through four additional single-cell cohorts. Groups of tumor cells were determined using the average values of the scores. Using cellphonedb, monocle, inferCNV, SCENIC, and Cytotrace, functional analyses were performed. Utilizing the RCTD approach, single-cell groupings were mapped onto spatial transcriptomics, analyzing cell dependency and pathway activity to distinguish between tumor cell subtypes. Differential expression analysis identified core genes in nucleotide metabolism, with single-cell and spatial transcriptomics analyses elucidating the function of these genes in tumor cells and the immune microenvironment. Prognostic models were developed from bulk transcriptome cohorts to forecast responses to immune therapy. Laboratory experiments were conducted to verify the biological function of the core gene.

**Results:**

Nucleotide metabolism is significantly elevated in tumor cells, dividing them into two groups: NUhighepi and NUlowepi. The phenotype NUhighepi was discerned to exhibit pronounced malignant attributes. Utilizing the analytical tool stlearn for cell-to-cell communication assessment, it was ascertained that NUhighepi engages in intimate interactions with fibroblasts. Corroborating this observation, spatial transcriptome cell interaction assessment through MISTy unveiled a particular reliance of NUhighepi on fibroblasts. Subsequently, we pinpointed NME1, a key gene in nucleotide metabolism, affirming its role in thwarting metastasis via in vitro examination. Utilizing multiple machine learning algorithms, a stable prognostic model (NRS) has been developed, capable of predicting survival and responses to immune therapy. In addition, targeted drugs have been identified for both high and low scoring groups. Laboratory experiments have revealed that NME1 can inhibit the proliferation and invasion of CRC tumor cells.

**Conclusion:**

Our study elucidates the potential pro-tumor mechanism of NUhighepi and the role of NME1 in inhibiting metastasis, further deepening the understanding of the role of nucleotide metabolism in colorectal cancer, and providing valuable targets for disrupting its properties.

**Supplementary Information:**

The online version contains supplementary material available at 10.1186/s12967-024-05495-y.

## Introduction

Colorectal cancer, recognized as one of the most prevalent malignancies [1], has been the subject of extensive research probing its pathogenesis. KRAS is a commonly mutated oncogene in colorectal cancer (CRC), with mutations present in approximately 40% of CRC cases. These mutations result in the constitutive activation of the KRAS protein, which acts as a molecular switch continuously stimulating downstream signaling pathways involved in cell proliferation and survival, thereby leading to tumorigenesis [2, 3]. Correspondingly, numerous therapeutic approaches targeting cancerous cells have been devised. Nevertheless, cell-based therapies for tumors persistently face multifaceted challenges such as tumor heterogeneity, metastasis, and treatment resistance, warranting continued investigation [4]. Consequently, a tailored examination of the pathogenesis of tumor epithelial cells becomes critically significant.

In an array of malignancies, nucleotide metabolism undergoes enrichment to satisfy the demands of unrestrained and expedited self-proliferation [5]. Concurrently, escalated nucleotide metabolism can induce genomic instability, thus contributing to further carcinogenesis [6]. The well-characterized oncogene, C-myc, masterminds nucleotide biosynthesis by augmenting the expression of various metabolic enzymes, including Dihydrolactase complex (CAD), Thymidylate synthase (TS), and Inosine monophosphate dehydrogenase (IMPDH) [7–9]. Strikingly, the modulation of nucleotide metabolism may directly alleviate immune suppression, as evidenced by secreted purines binding inhibitory receptors on immune cells [10]. Furthermore, studies have revealed [11, 12] that supplementary agents targeting nucleotide metabolism might evoke synergistic effects in conjunction with immunotherapy for cancer abatement. Consequently, downregulation of nucleotide metabolism emerges as a promising tactic to annihilate cancer cells or augment the efficacy of cancer treatments.

While the interplay between tumors and immune cells has undergone exhaustive scrutiny, the heterogeneous nature of tumor cells obscures the precise identification of these entities, necessitating further exploration. In the current investigation, we employed multiomics strategies, encompassing scRNA-seq and Spatial transcriptome (ST), to thoroughly dissect the communication between tumor and immune cells within colorectal cancer. We directed particular attention towards the function of tumor cells exhibiting elevated nucleotide metabolism (NUhighepi), a previously underexplored area, as identified within colorectal cancer tissues. We discerned that the TME modified the biological comportment of tumor-resident NUhighepi, rendering them pro-tumor proactive. A meticulous analysis also unveiled, for the inaugural time, a close localization between NUhighepi and tumor-associated fibrogenesis. We corroborated that this interaction accentuates the aggressive phenotype of NUhighepi by initiating pro-tumor signaling cascades in fibroblasts, such as the COL1A1/COL1A2_ITGB1 axis, thereby stimulating malignant transformation. Thus, these cellular dialogues may offer an optimal approach for colorectal cancer intervention. Simultaneously, we pinpointed NME1, an essential gene governing nucleotide metabolism, as a transfer inhibitor, with NME1 + epi aligning with the functional analysis of NUhighepi. This sheds novel light on the role of nucleotide metabolism in the trajectory of tumorigenesis progression.

## Methods and materials

### Data sources

In the context of this investigation, an amalgamation of four distinct and publicly available datasets was undertaken. These encompassed single-cell RNA sequencing data (GSE132257, EMTAB8107, GSE132465, GSE144735, GSE200997) [13], spatial transcriptomics information [14], along with the bulk transcriptomics drawn from the TCGA cohorts COAD and READ (https://xena.ucsc.edu) [15]. Genes correlated with nucleotide metabolism have been retrieved from the Gene Set Enrichment Analysis (GSEA) database.

### Single‑cell RNA sequencing analysis

The scRNA data was processed utilizing the R (v4.0.5) package Seurat (v4.0.2) [16]. The ensuing quality criteria were imposed: (1) genes discerned in fewer than 3 cells were omitted; (2) cells revealing fewer than 50 genes in total were disregarded; (3) cells in which more than or equal to 5% of genes were expressed in mitochondria were excluded. As quality control of the dataset had previously been undertaken in antecedent studies, no additional filtration of the scRNA-seq data was deemed necessary. The SCTransform method facilitated the normalization of the data, while the harmony method (v0.1.0) served to ameliorate batch effects and amalgamate the Seurat objects into an integrated dataset [17]. Principal Component Analysis (PCA) was employed to diminish the data dimensions. Methods such as FindNeighbors and FindClusters were harnessed to classify cells with analogous attributes. The calculation of cell-cycle scores was conducted via Seurat’s CellCycleScoring function, in light of observed cell cycle phase effects. For the purpose of data visualization, the Uniform Manifold Approximation and Projection (UMAP) algorithm was deployed.

### Cell type recognition

A differential expression analysis was performed across all genes contained within cell clusters. This was accomplished using Seurat’s FindAllMarkers function to discern the marker genes intrinsic to each cluster [16]. Criteria such as an adjusted P-value less than 0.05, expression percentage exceeding 0.25, and the absolute value of the log2 of the fold change (FC) greater than 0.25 were established as thresholds for the identification of marker genes (Supplementary Table 1). Following this, disparate cell clusters were ascertained and annotated via the singleR package, predicated upon the composition patterns of the marker genes. These determinations were then subject to manual verification and rectification, corroborated by the CellMarker database.

### Genetset score

A confluence of 10 distinct scoring methodologies was implemented through the use of irGSEA (https://github.com/chuiqin/irGSEA), predicated upon single-cell datasets. The specific parameters utilized in the irGSEA.score function were as follows: min.cells = 3, min.feature = 0, custom = F, msigdb = T, species = “Homo sapiens”, kcdf = ‘Gaussian’.

### Cell-cell communication analysis

In a quest to unearth potential interconnections amongst various cell types within the Tumor Microenvironment (TME), an intricate cell-cell communication analysis was undertaken utilizing CellPhoneDB. This is a comprehensive and publicly accessible compendium of meticulously curated receptors, ligands, and their subsequent interactions [18]. The analysis was executed using the CellPhoneDB Python package (2.1.7). Enriched receptor-ligand interactions between distinct cell types were extrapolated, contingent upon the expression of a receptor by one cell type juxtaposed against the expression of the corresponding ligand by another. This facilitated the identification of highly pertinent, cell type-specific interrelations between ligands and receptors, taking into consideration only those receptors and ligands articulated in more than 10% of the cells within the corresponding subclusters.

### Constructing single-cell trajectories in CRC

Utilizing the R package Monocle2 (v2.16.0), single-cell trajectory analyses were conducted under the supposition that a unidimensional ‘time’ parameter could elucidate the high-dimensional expression values, thereby uncovering cell-state transitions [19]. Clusters designated as Epithelial cells were integrated into the R environment, followed by the deployment of the newCellDataSet function to formulate an object, with the parameter expressionFamily = negbinomial.size. During the trajectory analysis, genes adhering to thresholds of mean_expression ≥ 0.1 and dispersion_empirical ≥ 1 * dispersion_fit, as discerned by Monocle2, were utilized to categorize cells in a pseudo-time sequence. The reduceDimension() function, with parameters reduction_method = “DDRTree” and max_components = 2, was applied to condense dimensions, while visualization functions such as ‘plot_cell_trajectory’ were employed to depict the minimum spanning tree on cells. Genes that exhibited variations in alignment with the pseudotime were calculated (q-val < 10^−5) by the “differentialGeneTest” function and visually represented through the plot_pseudotime_heatmap. Additionally, these genes were segregated into subgroups in accordance with the gene expression patterns.

### Single-cell copy-number variation (CNV) evaluation

The assessment of CNV within individual cells was meticulously executed utilizing the infercnv R package (version 1.4.0; https://github.com/broadinstitute/inferCNV/wiki) [20], with specific focus on the CNVs of Epithelial cells. The inferCNV analysis was initiated with parameters encompassing “denoise,” default hidden Markov model (HMM) configurations, and a threshold value of 0.1 for “cutoff.” In a concerted effort to attenuate the occurrence of spurious CNV identifications, the default Bayesian latent mixture model was employed to determine the posterior probabilities of CNV alterations in each cell, utilizing a default threshold value of 0.5.

### The regulon activity of TFs with SCENIC

The SCENIC algorithm was ingeniously crafted to scrutinize the regulatory network in connection with Transcription Factors (TFs) and unearth regulons, comprised of TFs and their affiliated target genes. A log-normalized expression matrix with gene identifiers arranged in rows and cells in columns, generated via Seurat, served as the input to SCENIC (version 1.2.4) [21]. Subsequently, a motif dataset (hg19-tss-centered-10 kb-7species.mc9nr.feather) was harnessed to architect regulons for each TF within SCENIC. The co-expressed genes associated with each TF were constructed with the aid of GENIE3 software, succeeded by Spearman’s correlation between the TF and the potential targets. Thereafter, the “runSCENIC” procedure facilitated the construction of Gene Regulatory Networks (GRNs), also referred to as regulons. The culmination of this process was the analysis of regulon activity via AUCell (Area Under the Curve) software, applying a default threshold to binarize specific regulons, whereby “0” symbolized the “off” state of TFs, and “1” denoted the “on” state.

### CytoTRACE analysis

Engineered by Gulati et al., the CytoTRACE algorithm is a sophisticated technique designed to capture, streamline, and quantify the expression levels of genes exhibiting the highest correlation with single-cell gene counts within scRNA-Seq data. Upon completion of the CytoTRACE algorithm’s computation, each singular cell is endowed with a score epitomizing its stemness within the prescribed dataset. CytoTRACE, serving as a robust computational framework, excels in predicting differentiation states through scRNA-seq data and has been substantiated in large-scale datasets, superseding pre-established computational methodologies of stemness evaluation [22]. The R package CytoTRACE v0.3.3 was leveraged to compute the CytoTRACE scores for malignant cells, wherein scores ranging from 0 to 1 were assigned, with elevated scores indicative of heightened stemness (lesser differentiation), and conversely, diminished scores reflecting lower stemness.

### Spatial transcriptomics data analysis

The Seurat R package was employed for the handling and illustration of spatial transcriptomics data (ST). To standardize the ST data, the SCT technique was utilized, and the functions SelectIntegrationFeatures, PrepSCTIntegration, FindIntegrationAnchors, and IntegrateData were engaged to amalgamate the ST data. Following this, an unsupervised clustering approach was employed to aggregate analogous ST locales. Annotations for cell populations were grounded on both hematoxylin and eosin staining (HE) sections and the markedly variable genes within each cluster. The functions SpatialDimPlot and SpatialFeaturePlot were synergistically used to delineate the expression levels of cells within the ST data.

### Cell type decomposition analysis of spatial transcriptome data

The technique of Robust Cell Type Decomposition (RCTD) was invoked to correspond the identified cell types within the reference scRNA-seq dataset to the spatial transcriptomic data [23]. Distinctive marker genes for every cell type were ascertained via the Seurat function FindAllMarkers, restricting the scope to markers exhibiting positive log2-transformed fold alterations. Subsequently, the standard RCTD analysis pathway was rigorously adhered to, focusing on both the reference and Visium spatial transcriptomics data in a doublet mode set to full.

### Estimation of functional information from spatial data

For individual locales, we approximated signaling pathway engagements utilizing PROGENy’s [24, 25] model matrix (v1.12.0), capitalizing on the leading 1,000 genes of each transcriptional footprint and the sctransform normalized data.

### Spatial map of cell dependencies

The implementation of MISTy [26] in mistyR (v1.2.1) was employed to assess the significance of the abundance of each primary cell type in elucidating the prevalence of other major cell types. Cell-type RCTD estimates across all slides were fashioned into a multifaceted model utilizing three discrete spatial contexts: (1) an intrinsic perspective that evaluates correlations among the deconvolution estimations within a specific locale, (2) a juxta perspective that accumulates the observable deconvolution estimations of proximate neighbors (maximum distance threshold = 5), and (3) a para perspective that ascribes weight to the deconvolution estimations of more remote neighbors of each cell type (effective radius = 15 spots). The consolidated estimated standardized importances (median) of each perspective across all slides were construed as dependencies between cell types in varying spatial contexts, such as colocalization or mutual exclusion. However, these observed interactions were devoid of implications of any causative relationships. Prior to aggregation, the importances of all predictors of target cell types with an R² value below 10% were omitted for each slide.

In an effort to correlate tissue structures with functional attributes, a MISTy model was constructed to delineate the distribution of PROGENy’s standardized pathway activity scores. This encompassing multi-view model encompassed the following predictors: (1) an intrinsic perspective to model pathway interplay within a locale, (2) a juxta perspective to model pathway interaction among neighboring spots (maximum distance threshold = 5), (3) a para perspective that estimates pathway correlations within expansive tissue structures (effective radius = 15), (4) an intrinsic perspective, and (5) a para perspective encompassing RCTD estimations (effective radius = 15). The latter two perspectives are explicitly crafted to model the connections between cell-type compositions of spots and pathway activities. Notably, cycling cells and TNF were excluded from the aforementioned analyses. Prior to aggregation, the importances of all predictors of targeted pathway activities with an R² value under 10% were discarded for each slide.

### Spatial trajectory analysis and cell-cell interaction

or individual locales, Cell-Cell interaction was estimated through stLearn [27], followed by an adaptation of pseudo-time trajectory analysis in space using stLearn, which harnessed PAGA trajectory analysis founded on tissue-wide SME normalized gene expression data to uncover connections within subclusters. To model the cancerous progression among the sections, a pseudotime spatial trajectory algorithm was employed, discerning both spatial and transcriptional linkages amid the sub-clusters.

### Survival analysis

To investigate the specific role of distinct cellular entities in clinical diagnostics and prognostic evaluation, we utilized Survival (v3.2-10) and Survminer (v0.4.9) to orchestrate survival analysis on the COAD and READ cohorts. The infiltration of cell populations (comprising the top 20 differentially expressed genes as identified in scRNA-seq) was computed through the ssGSEA algorithm. The median was elected as the demarcation point to segregate patients into differentiated categories (either high or low). The Kaplan–Meier survival curve was subsequently synthesized utilizing the survfit function.

### Cell culture and transfection conditions

SW480 and HCT116 cells were provided by the public laboratory of Tianjin Medical University Cancer Hospital and cultivated in a meticulously regulated environment of 5% CO₂ at 37 °C, thriving in DMEM medium (Boster, China) augmented with 10% FBS (HyClone, USA). In a transient maneuver to suppress the expression of NME1, siRNAs (GenePharma, Suzhou, China) were transfected into SW480 and HCT116 cells via Lipofectamine 3000. The particular siRNA sequences targeting NME1 in SW480 and HCT116 cells were implemented as follows: siRNA negative control (NC) (5′-UUCUCCGAACGUGUCACGUTT-3′), siRNA-NME1 #1 (5′-CCCUGAGGAACUGGUAGAUTT-3′), and siRNA-NME1 #2 (5′-GCUGUAGGAAAUCUAGUUATT-3′).

### qPCR and western blotting (WB)

Real-time PCR was conducted according to previously outlined protocols [9]. The primers designated for RT-PCR were conceived within our laboratory and synthesized by Sangon (Shanghai, China, Supplementary Table 2). Cells were disrupted in an ice-cold lysis buffer replete with phosphatase and protease inhibitors. Utilizing the bicinchoninic acid assay, protein concentrations were ascertained. Protein samples were fractionated by a 4–12% SDS/PAGE gradient and subsequently transferred onto PVDF membranes. These membranes were subjected to a blocking process and incubated with both primary and secondary antibodies. The immunoreactive proteins were visualized through the application of a chemiluminescent solution.

### Transwell assays and wound healing

Migration capabilities were assessed employing the Boyden chamber assay, featuring an 8-µm pore size. CRC cells were suspended in 200 µL of FBS-free medium (1 × 10⁵ cells) and introduced to the upper chamber (BD, USA). Medium enhanced with 10% FBS was introduced into the lower chamber. Following a 24-hour incubation, cells were stabilized and stained, with the quantity of cells in six arbitrarily chosen fields tallied under microscopic examination. For the wound healing procedure, the transfected cells were sown into six-well plates at a concentration of 1 × 10⁵ cells per well. Upon reaching a confluence exceeding 90%, a linear wound was artfully created with a sterile pipette tip. The cells were subsequently incubated in a serum-free medium for 24 h. After rinsing twice with PBS, the migration process was closely monitored under a microscope. The migratory prowess of the cells was quantitatively measured in terms of wound width rate, with relative migration calculated as (the scratch width at 24 h divided by that at 0 h) × 100%.

### Machine learning algorithms

This comprehensive model encompassed a diverse array of algorithms, including Random Survival Forests (RSF), Elastic Net (Enet), Lasso, Stepwise Cox, Ridge, CoxBoost, Partial Least Squares Regression for Cox Models (plsRcox), Supervised Principal Component (SuperPC), Gradient Boosting Machine (GBM), and Survival Support Vector Machine (survival SVM). These algorithms were applied in different configurations to identify the model exhibiting the highest mean concordance index (C-index) across all validation datasets. The accuracy of the Risk score was validated through the computation of the Area Under the Curve (AUC) using the ‘timeROC’ package. Additionally, the Risk score as an independent predictive factor was corroborated through Cox regression analysis, conducted with the ‘survival’ package in R.

### Immunoinfiltration assessment

The comparative analysis of immune checkpoint distribution across these subgroups was conducted, utilizing the ESTIMATE R package to ascertain the immune/stromal score of the neoplastic tissue. Furthermore, the DNA methylation score of the tumor-infiltrating lymphocytes (MeTIL) was meticulously computed in adherence to established protocols. Additionally, the assessment of the enrichment of 24 distinct varieties of cells within the tumor immune microenvironment was executed through Gene Set Variation Analysis (GSVA).

### Immunotherapy efficacy prediction

The cohorts for immunotherapy analysis were sourced from the TIGER database [53], encompassing datasets GSE91061, phs000452, and PRJEB23709. Single cell immunotherapy cohort (GSE120575) was downloaded from GEO (https://www.ncbi.nlm.nih.gov/geo/query/acc.cgi?acc=GSE120575).

### Drug prediction

Drug sensitivity data of CCLs were achieved from the Cancer Therapeutics Response Portal (CTRP v.2.0, released October 2015, https://portals.broadinstitute.org/ctrp) and PRISM Repurposing dataset (19Q4, released December 2019, https://depmap.org/portal/prism/).

## Results

### Nucleotide metabolism is increased in tumor epithelial cells

**Fig. 1:**
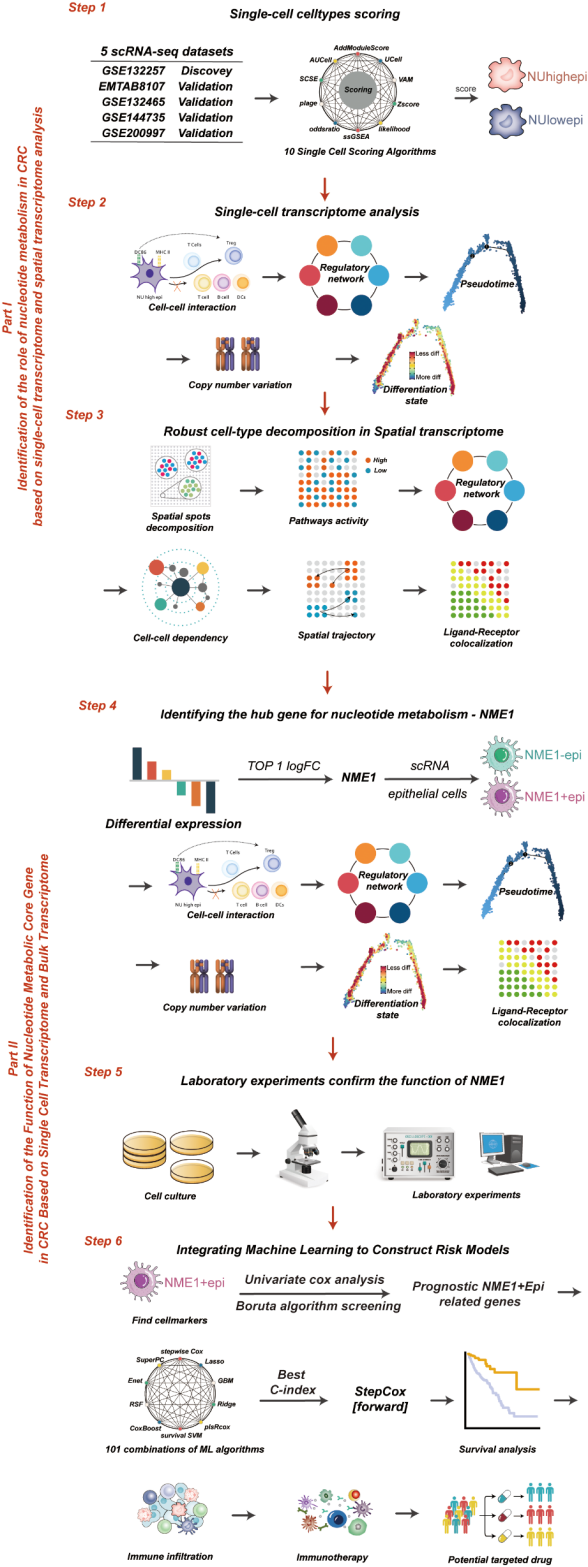
The workflow of the study

**Fig. 2:**
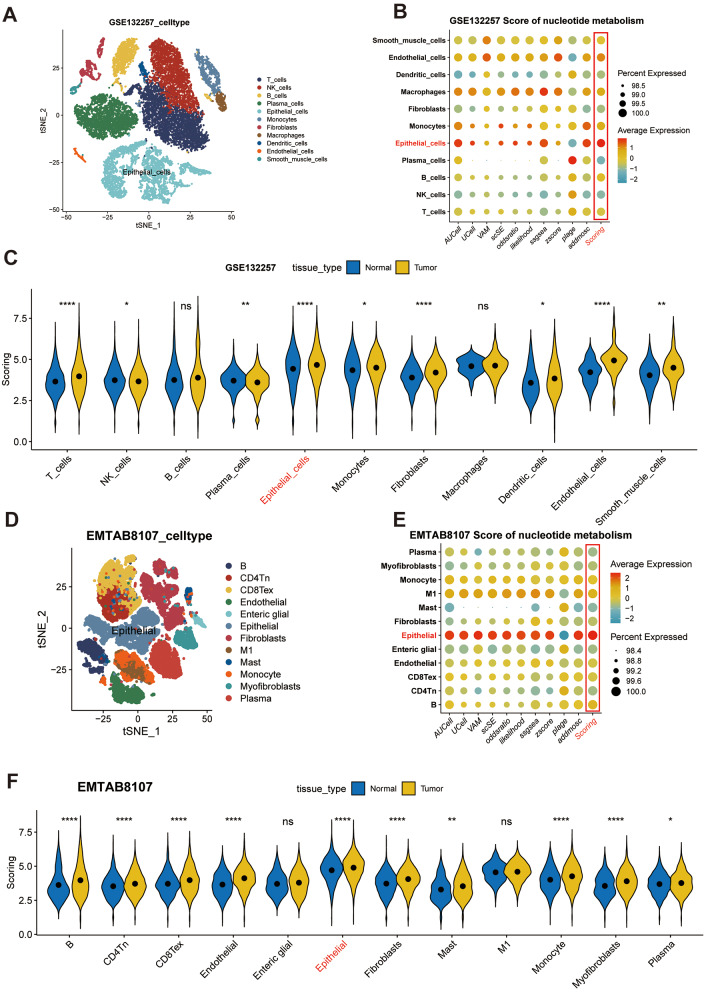
Nucleotide metabolism is increased in tumor epithelial cells: (**A**) T-distributed stochastic neighbor embedding (tSNE) plots of the colon cancer cells, colored by cell type in GSE132257. (**B**) Bubble plot of multi-methods of Nucleotide metabolism score in diverse celltype in GSE132257. (**C**) Violin plots of the scoring, faceted by tissue types in GSE132257. (**D**) T-distributed stochastic neighbor embedding (tSNE) plots of the colon cancer cells, colored by cell type in EMTAB8107. (**E**) Bubble plot of multi-methods of Nucleotide metabolism score in diverse celltype in EMTAB8107. (**F**) Violin plots of the scoring, faceted by tissue types in EMTAB8107. ****P < 0.0001, ***P < 0.001, **P < 0.01, *P < 0.05, ns *P* > 0.05

The process of our investigation is illustrated in Fig. 1. This investigation encompassed a selection of specimens, including both neoplastic and peri-neoplastic tissues, from two individuals suffering from colorectal carcinoma. Directed by the insights gleaned from the CellMarker database in conjunction with the SingleR package, 11 fundamental cellular categories were identified, encompassing T cells, NK cells, B cells, Plasma cells, Monocytes, Macrophages, Dendritic cells, Fibroblast cells, Smooth muscle cells, Endothelial cells, and Epithelial cells (Fig. 2A). The distinctive cellular markers, defined by variations within these principal cellular classifications, were elucidated via a bubble plot (Supplementary Fig. 1A), while the heterogeneous cellular proportions and quantities particular to each patient were portrayed in Supplementary Fig. 1B. Fascinatingly, when examining the origins of these tissues, it was revealed that immune cells, notably T cells, Monocytes, and Macrophages, were markedly concentrated within the tumor tissues.

An augmenting body of preclinical data has corroborated that the anomalous metabolism of malignant cells fosters immune evasion by altering the metabolic constitution of the tumor microenvironment (TME) [28]. Emerging evidence suggests that the manipulation of nucleotide modification impairs the anticancer effectiveness of T cells [29], macrophages [30], innate immune cells [31], and dendritic cells [32] within the TME.

To meticulously examine nucleotide metabolism in colorectal cancer, we employed ten single-cell scoring techniques to compute the nucleotide metabolism scores, then computed the average of these ten scores, termed “Scoring.” It was revealed that the epithelial cells demonstrated the most pronounced Scoring, followed by endothelial cells (Fig. 2B). Additionally, the nucleotide metabolism index of epithelial cells within neoplastic tissue was perceptibly elevated in comparison to that within normal tissue (Fig. 2C). As per the dimensionality reduction diagram, the nucleotide metabolism rating of epithelial cells in non-cancerous tissues was preeminent, whereas those of epithelial cells, endothelial cells, smooth muscle cells, monocytes, and macrophages within neoplastic tissues were heightened (Supplementary Fig. 2C). The increased nucleotide metabolism score in epithelial cells was confirmed in four parallel single-cell cohorts (Fig. 2D-F, Supplementary Fig. 2A-F, Supplementary Fig. 3A-C). Building upon these scores, we segregated two clusters of epithelial cells with both high (NUhighepi) and low (NUhighepi) nucleotide metabolism, with the objective of probing deeper into the functional role of nucleotide metabolism within malignant cells.

### Function analysis of nucleotide metabolism in scRNA-seq data

**Fig. 3:**
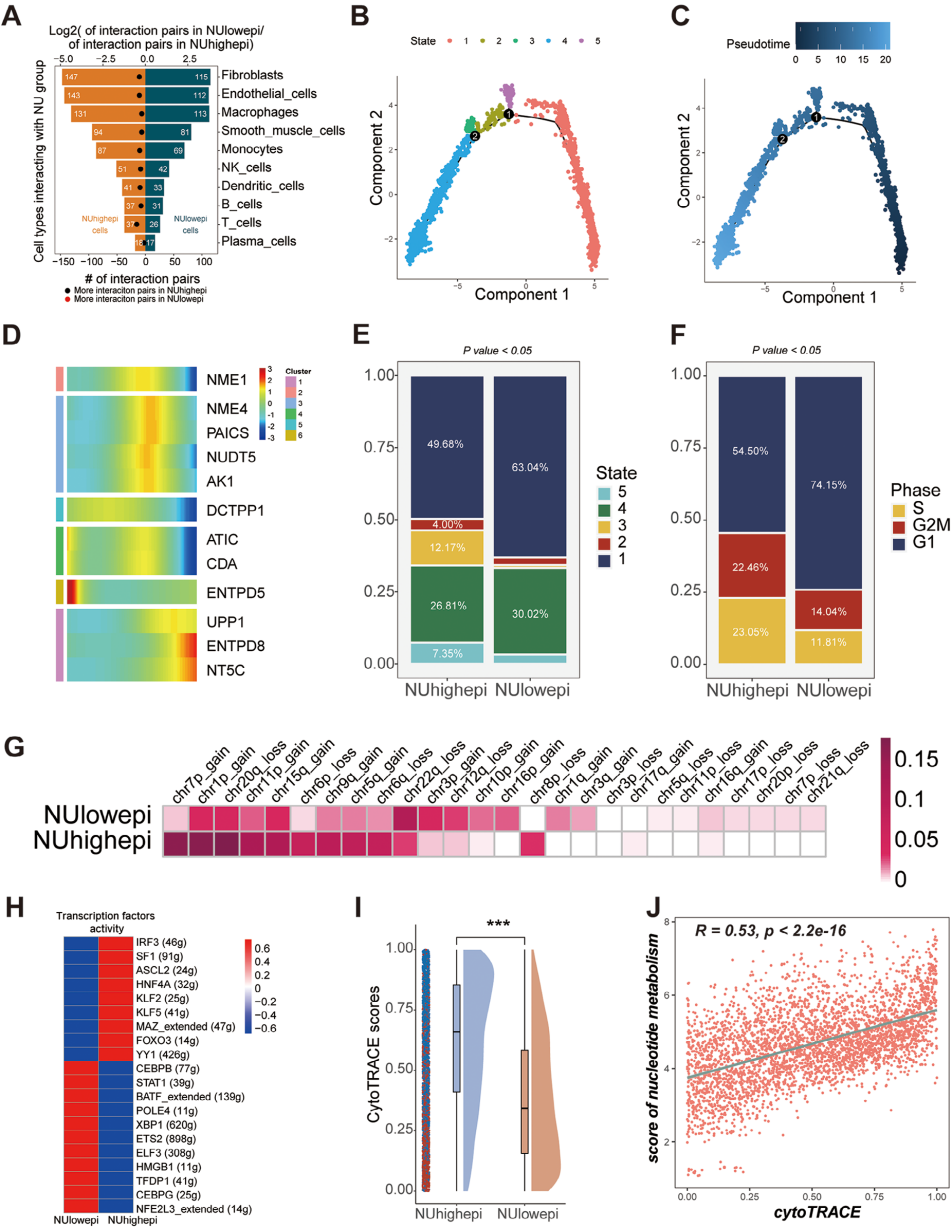
Function analysis of NUhighepi/NUlowepi in scRNA-seq data: (**A**) Barplot showed the number of other cell types communicating with NU group. (**B**-**C**) Pseudotime analysis of epithelial cells. (**D**) Heatmap showed pseudotime of Nucleotide metabolism related genes (NRGs). (**E**-**F**) Barplot showed pseudotime states (**E**) or cell-cycle states (**F**) of NU group. (**G**) Heatmap showed inferCNV profiles of NUhighepi and NUlowepi. (**H**) Heatmap showing transcriptional activity of NU group. Intensity of color indicates average transcription factors activity. (**I**) Raincloud plot of CytoTRACE scores by NU group in scRNA-seq data. The center of the box plot are median values, the bounds of the box are 25% and 75% quantiles. (**J**) Scatter plot of the correlation between CytoTRACE score and Nucleotide metabolism score. ****P < 0.0001, ***P < 0.001, **P < 0.01, *P < 0.05, ns *P* > 0.05

We executed a comprehensive examination of the interactions between NUhighepi/NUlowepi and other cellular entities by employing Cellphoedb, in conjunction with single-cell RNA sequencing (scRNA-seq) data. It was ascertained that the count of conceivable ligand-receptor dyads involving NUhighepi and other cells was substantially augmented compared to NUlowepi (Fig. 3A). The cells manifesting pronounced interplay were uniform, including fibroblasts, endothelial cells, and macrophages. Subsequently, we delved into the interactions between NUhighepi/NUhighepi and other cells via Cellphoedb, considering the NU group as either receptor or ligand, and stumbled upon commensurate results, with the exception that NUlowepi exhibited a marked liaison with plasma cells when operating as a receptor (Supplementary Fig. 4A-B). By contrasting the communicative signals with NUlowepi, we detected that the signaling conduit displaying vigorous interaction between NUhighepi and fibroblasts consisted of the PLA2G2A_a5b1 complex, MIF_TNFRSF14, and TFRC_TNFSF13B (Supplementary Fig. 4C), whilst the pathway connecting NUhighepi and endothelial cells entailed the PLA2G2A_a5b1 complex, MIF_TNFRSF10D, and MIF_TNFRSF14 (Supplementary Fig. 4D), and the route between NUhighepi and macrophages incorporated the PLA2G2A_a4b1 complex, PLA2G2A_a5b1 complex, and TFRC_TNFSF13B (Supplementary Fig. 4E). Simultaneously, we discerned that the signal exemplifying the most formidable interaction between NUhighepi and other immune cells was predominantly the PLA2G2A_a5b1 complex, barring the signal with macrophages, which was MIF_TNFRSF14 (Supplementary Table 3). When NUhighepi/NUlowepi were designated as receptors, the signaling channels with other cells were chiefly related to MIF (Supplementary Fig. 4F-H). We discovered through cell communication analysis that NUhighepi has stronger interactions with stromal cells (fibroblasts, endothelial cells), primarily facilitated by PLA2G2A-related complexes.

Additionally, we exploited our scRNA-seq data to investigate the dynamics of NRGs’ (nucleotide metabolism-related genes) expression patterns at a single-cell resolution using Monocle analysis for trajectory inference (Fig. 3B-C). We unearthed that genes manifest during intermediate and advanced stages encompassed ATIC, DCTPP1, NME1, NUDT5, NME4, and PAICS, whereas CDA, AK1, UPP1, ENTPD8, and ENTPD5 were operative during the initial phase (Fig. 3D). Pseudotime analysis divulged that NUhighepi displayed an increased fraction of states 3 and 5, reflective of intermediate and advanced stages (Fig. 3E), and cell-cycle scrutiny revealed an augmented proportion of S and G2M phases in NUhighepi, denoting intensified progression activity (Fig. 3F). Through an exploration of the evolution of epithelial cell subclusters during oncogenic progression, we pinpointed a significant escalation in the copy number variance scores of NUhighepi across chr1p_gain, chr7p_gain, chr8q_gain, chr13q_gain, chr13q_loss, chr19q_gain, and chr22q_gain (Fig. 3G, Supplementary Fig. 5A). Furthermore, cell-type specific regulators correlated with immune responses and/or tumorigenesis or metastasis, such as CEBPB, STAT1, BATF, HMGB1, TFDP1, and POLE4, were distinctly enriched in NUhighepi, as ascertained by the Regulon Specificity Score (Fig. 3H). Through CytoTRACE assessment, it was found that compared to NUlowepi, the tumor stemness characteristics of NUhighepi significantly increased (Fig. 3I), and there is a positive correlation between nucleotide metabolism enrichment scores and CytoTRACE scores (Fig. 3J). Concurrently, we also identified nucleotide metabolism-related genes (NRGs) that were differentially manifested between tumorous and normal epithelial cells (Supplementary Table 4, Supplementary Fig. 5B). Specifically, the expression of NME1 was observed to be significantly augmented within tumorous epithelial cells in comparison to their normal counterparts. Through transcription factor regulatory network and cell stemness analysis, we found that NUhighepi exhibits stronger stemness activity and the activity of transcription factors related to tumor invasion.

### Spatial transcriptomics to identify nucleotide metabolism in CRC

**Fig. 4:**
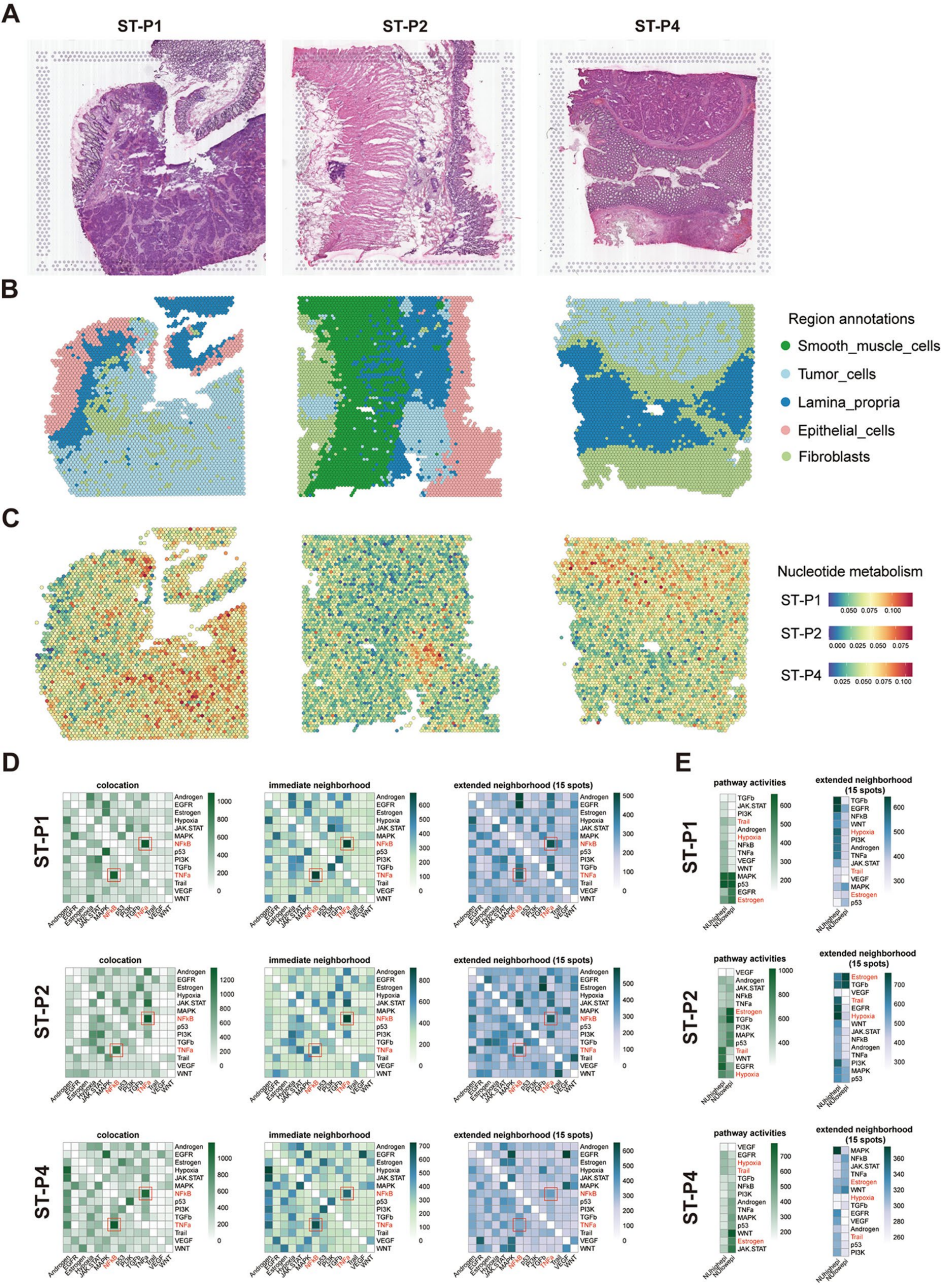
Spatial transcriptome analysis of NUhighepi/NUlowepi: (**A**-**B**) Spatial images show the tissue architecture of CRC inferred by unsupervised clustering method. (**C**) Featureplot of Nucleotide metabolism in spatial organization. (**D**) PROGENy pathway activities within a spot, and in the immediate or extended neighbourhood on the prediction of pathway activities inferred from spatially contextualized models. (**E**) Median standardized importances (> 0) of cell-type abundances within the spot and in the extended neighbourhood (effective radius = 15)

In the present investigation, attention was directed towards the spatial transcriptomics data of three patients, stemming from an antecedently published colorectal cancer (CRC) study (ST-P1, ST-P2, and ST-P4) [14]. Drawing upon the hematoxylin and eosin (HE) stained sections and differentially expressed genes (DEGs) for each cluster, we identified and cataloged the spatial locations into five predominant clusters: neoplastic cells, fibroblasts, smooth muscle cells, epithelial cells, and lamina propria (Fig. 4A-B). A marked accumulation of nucleotide metabolism was discerned within the neoplastic region (Fig. 4C). To systematically classify the composite cell types present in each spot, we employed the RCTD method, juxtaposing spatially resolved transcriptomic data with single-cell RNA sequencing (scRNA-seq) data. In untreated specimens, a consistency in epithelial cell patterning was observed, congruent with scRNA-seq, manifesting as NUhighepi’s pronounced enrichment in tumor aera (Supplementary Fig. 6A). Subsequently, we approximated the activities of signaling pathways utilizing PROGENy (Methods) for each spot derived from the spatial gene expression data. This juxtaposition of spatially confined pathway activities with the assessed cellular abundance per spot afforded us the ability to correlate spatial cell composition with cellular function on an individual slide basis. Within regions replete with neoplastic cells, increased activities of EGFR, Hypoxia, MAPK, and TGFβ signaling pathways were discerned (Supplementary Fig. 6B). Utilizing SCENIC, we inferred the spatial distribution of transcription factor activity within various regions, discovering a heightened activity of MYC within neoplastic tissue sections, an observation in alignment with preceding research [7–9] (Supplementary Fig. 6C). The spatial transcriptomics pathway activity analysis and transcription factor analysis further confirmed that NUhighepi exhibits stronger stemness activity and higher activity of tumor invasion-related transcription factors.

We subsequently examined whether the abundances of principal cell types within specific spots could be prognosticated by their spatial context, as defined by the cellular compositions of their immediate vicinities. Three distinct neighborhood area sizes were assessed using MISTy, encompassing: (1) the intrinsic significance of cell-type abundances within a particular spot (colocalization), (2) within the proximal neighborhood (a radius encompassing 5 spots), and (3) within an expansive neighborhood extending to a radius of 15 spots. We discerned that NUhighepi cells were indicative in the abundance of Plasm cells, NK cells, Fibroblasts, Endothelial cells, and Smooth muscle cells across all spots, likely mirroring interdependencies between immune cells and epithelial cells (Supplementary Fig. 7A). Particularly salient were the observed robust dependencies between NUhighepi cells and Plasm cells, which were markedly co-enriched in ST-P1 (Supplementary Fig. 7A), consonant with the well-established role of nucleotide metabolism in the nuanced variation of the tumor immune microenvironment. To forge a connection between tissue organization and its functional aspects, we undertook an analysis of spatial interdependencies between signaling pathways and cell types. The modeled significance of colocalized pathways revealed intricate relationships between NFkB and TNFa signaling (Fig. 4D), manifesting a mutual affinity in spatial distribution (Supplementary Fig. 4B). Both pathways were correlated with the abundance of either NUhighepi or NUlowepi (Fig. 4D). NFkB signaling was found to foster colorectal cancer cell proliferation [33], whereas TNF-α instigated the PI3K/Akt signal transduction pathways, subsequently stimulating the downstream NF-κB pathway p65, culminating in the augmentation of metastatic capabilities in colon cancer cells [34]. The spatial conjunction of these NUhighepi or NUlowepi-associated pathways underscores the functional heterogeneity within colon cancer cells. We further detected colocalized and extended neighborhood correlations of known pivotal pathways in tumor cells, including Trail and Hypoxia as predicted by NUhighepi, and Estrogen as predicted by NUlowepi (Fig. 4E). Through cell and pathway dependency analysis, it was found that NUhighepi has a good colocalization relationship with stromal cells (Fibroblasts, Endothelial cells, and Smooth muscle cells). Additionally, it was found that NFkB and TNFa pathways have a good dependency, suggesting a synergistic relationship between these pathways in the development and progression of colorectal cancer. Furthermore, NUhighepi seems to be more dependent on the Trail and Hypoxia signaling pathways.

We refined the analysis of cell-cell interactions within spatial contexts employing stlearn, a method that leverages spatial information in conjunction with gene expression profiles to pinpoint precise locations within tissue where elevated ligand-receptor interaction activity coincides with diverse cellular co-localization. Our examination revealed that the foremost rankings of Ligand-Receptors (LRs), specifically COL1A1_ITGB1 and COL1A2_ITGB1, were profusely enriched in the NUhighepi region (Supplementary Fig. 8A-B, Supplementary Table 5). Gene Ontology (GO) enrichment analysis illuminated that these preeminent Ligand-Receptors were particularly associated with cellular processes such as cell-substrate adhesion, wound healing, and further cell-substrate adhesion (Supplementary Fig. 8B, Supplementary Table 6). The Cell-Cell Interaction (CCI) Analysis discerned that the pairs COL1A1/COL1A2_ITGB1 constituted the more potent LRs within the interaction nexus between fibroblasts and both NUhighepi and NUlowepi. These findings denote that the spatial CCI dynamics of tumor cells were intricately related to fibroblasts, entities previously demonstrated to secrete deoxycytidine. Such secretion may contribute to a compensatory tumor nucleoside salvage pathway flux in the presence of inhibition pertaining to de novo synthesis in vivo [35] (Supplementary Fig. 8C). Spatial transcriptomics cell communication analysis confirmed the interaction between NUhighepi and stromal cells (fibroblasts), primarily mediated through the COL1A1/ COL1A2.

### The spatial trajectory inference in intra-tumoral

Within the tumor’s microenvironment, understanding the in vivo dynamics is crucial to deciphering the genesis and evolution of cancerous cells or clones. One theoretical construct that has gained traction in elucidating such dynamics is the pseudo-time concept, commonly harnessed in single-cell RNA sequencing (scRNA-seq) data interpretation. This notion is tailored to detect biological processes discernible from gradational shifts in transcriptional states across tissue substrates. For the classification of the amalgamated cell types within each spatial domain, we employed the RCTD methodology, juxtaposing spatially resolved transcriptomic data with its paired scRNA-seq data.

**Fig. 5:**
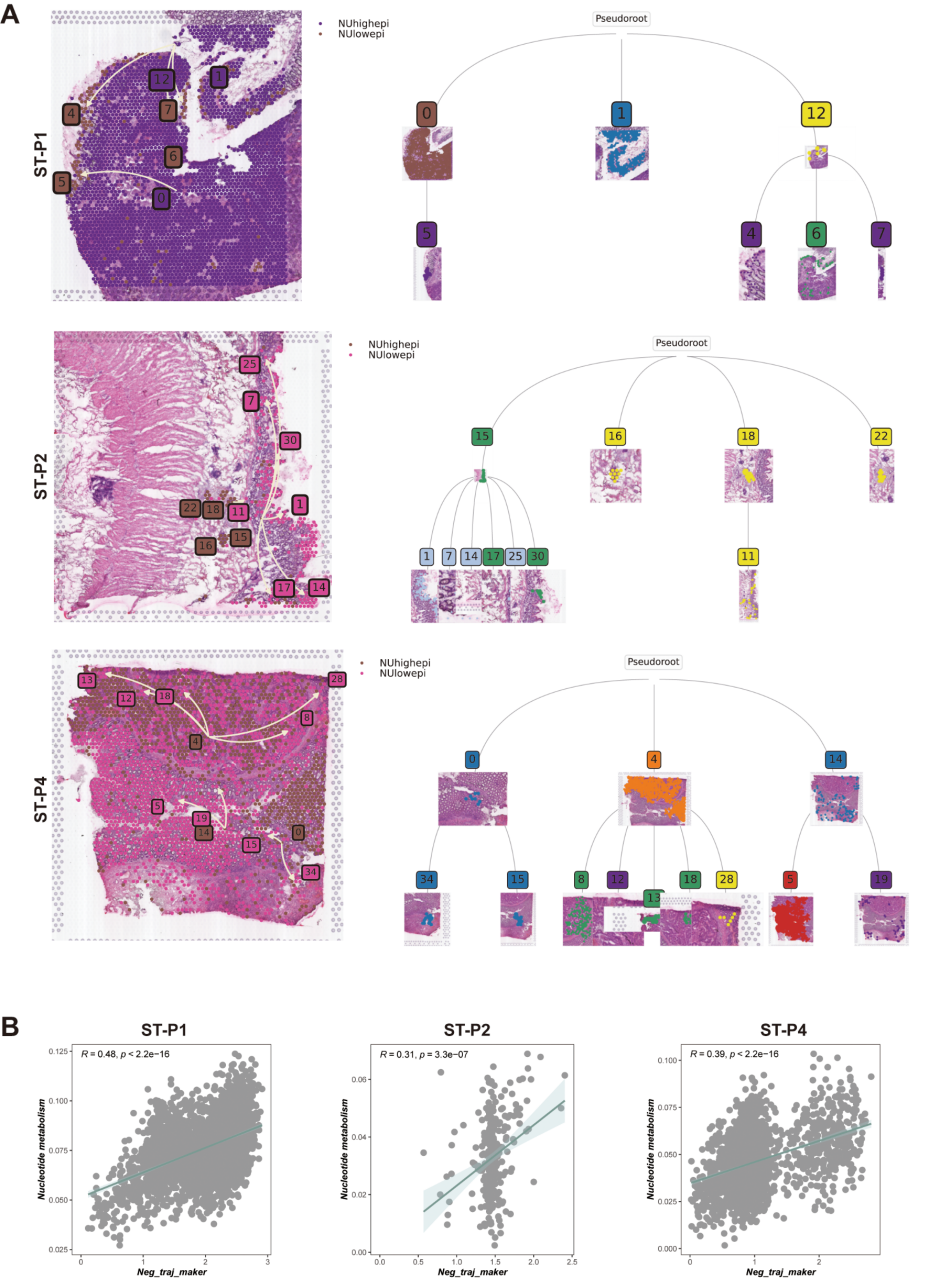
Spatial trajectory inference of NUhighepi/NUlowepi: (**A**-**B**) The pseudo-time spatial trajectory algorithm was applied to find the spatial evolution trajectory and transcriptional connections among NU group. (**C**) Nucleotide metabolism and negative spatial trajectory gene expression quantity association analysis in each spot of section

In both the global and local paradigms of spatial trajectory deduction, we incorporated the pseudotime spatial trajectory algorithm. This approach was instrumental in deciphering the spatial evolutionary trajectory and the intricate transcriptional linkages among the subclones within colon cancer (Fig. 5A). Our observations posited that the spatial trajectory directionality of tumor cells originates from a zone of heightened nucleotide metabolism, culminating in areas marked by reduced nucleotide metabolism. Moreover, clusters characterized by elevated nucleotide metabolism were concomitantly associated with increased metastatic tendencies. This relationship suggests that the evolutionary trajectory of subclones commences with subclones bearing heightened metastatic invasiveness, and over time, morphs into subclones with diminished invasive and metastatic capacities.

Utilizing spatial-pseudotime analyses, the progression trajectories of the subclones were discerned. This technique enabled the elucidation of an overarching hierarchical tree diagram, illustrating the global spatial trajectory inference across subclones (ST-P1, ST-P2, & ST-P4). For instance, ST-P2 manifested four distinct clades (15, 16, 18, 22), with a predominant clade branching extensively (15 − 1, 15 − 7, 15 − 14, 15–17, 15–25, 15–30, 18 − 11), emblematic of the primary evolutionary trajectories of tumor progression. Comparable patterns were detected in both ST-P4 and ST-P1, each evincing a major clade with a myriad of branches, encapsulating the divergent evolutionary trajectories inherent to subclone progression within colon cancer (Fig. 5A). Our subsequent analyses centered on determining trajectory-based differential gene expression. Here, genes on the left (colored red) showcased a negative correlation with the spatial trajectory, while those on the right (blue) displayed a positive correlation (Supplementary Fig. 8D-F).

To delve deeper into the determinants propelling the spatial trajectories of subclones, we amalgamated genes that were negatively correlated with the spatial trajectory into a cohesive gene set. After scoring these in relation to tumor-associated locations using myriad scoring techniques, our findings revealed a significant positive association between nucleotide metabolism scores and genes inversely related to spatial trajectories, with respective correlations in ST-P1, ST-P2, and ST-P4 being *R* = 0.48, *R* = 0.31, and *R* = 0.39 (Fig. 5B). Spatial transcriptomic evolutionary trajectory analysis confirmed that NUhighepi has the ability to differentiate into other tumor epithelial cells, suggesting that NUhighepi possesses a higher differentiation potential. Additionally, there is a positive correlation between nucleotide metabolism and genes related to differentiation and evolution, indicating that nucleotide metabolism can promote tumor initiation and progression.

### Spatial transcriptome and scRNA analyses uncover NME1 in CRC

**Fig. 6:**
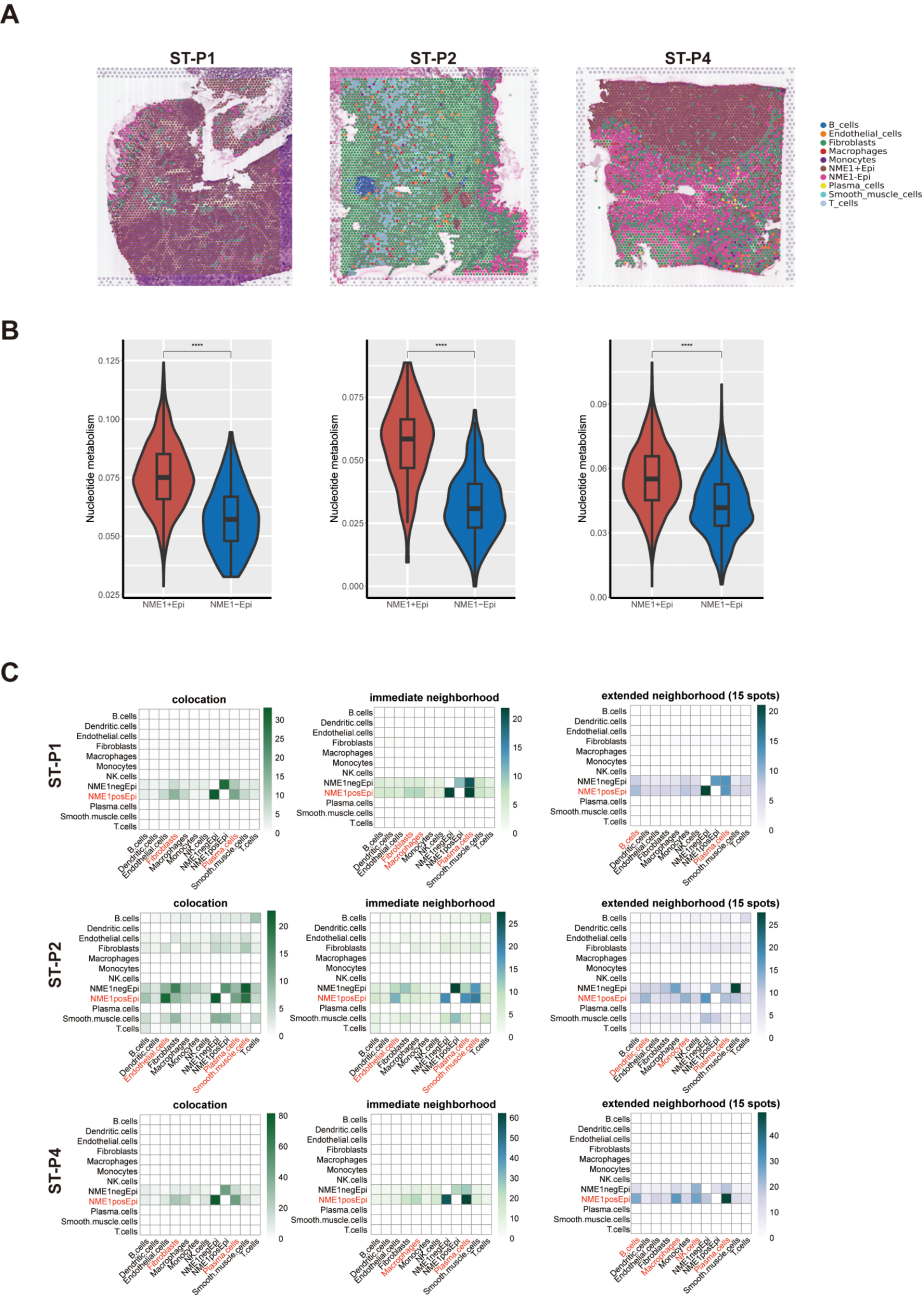
Spatial transcriptome analysis of NME1 group: (**A**) Cell types of each spatial spot revealed by RCTD. (**B**) Violin plot of nucleotide metabolism, grouped by NME1 group. (**C**) Median standardized importances (> 0) of cell-type abundances in the prediction of other cell types within the colocation (left part), immediate neighbourhood (medial part) and the extended neighbourhood (effective radius of 15 spots) (right part) inferred from spatially contextualized models. Cell-type abundances of colocation, the immediateand extended neighbourhood of NU group

We previously found that NME1 is the most significantly nucleotide metabolism-related gene in tumor epithelial cells (Fig. 3). Therefore, we divided the tumor epithelial cells in the single-cell data into NME1-positive (NME1 + Epi) and NME1-negative (NME1-Epi) cell groups to further explore the key mechanisms of nucleotide metabolism. We subsequently utilized the RCTD method to classify the various cell types inhabiting each spatial location (Fig. 6A). This endeavor was achieved by amalgamating spatially resolved transcriptomic data with scRNA-seq data, facilitating an exhaustive categorization of cell types at spatial resolution. Our examination revealed that the spatial nucleotide metabolism of tumor cells has been correlated with NME1 (Fig. 6B). We then proceeded to ascertain whether the prevalences of principal cell types within particular regions could be anticipated by their spatial context as delineated by the cell-type compositions of their proximate surroundings. Our observations indicated that NME1 + epi cells were prognostic in the prevalence of Plasm cells, Fibroblasts, Endothelial cells, and Smooth muscle cells within all localized areas, possibly mirroring interdependencies between immune and epithelial cells (Fig. 6C). It is salient that we discerned significant correlations between NME1 + epi and Plasm cells, demonstrating robust co-enrichment in ST-P1, ST-P2, and ST-P4. These insights corroborate the previously scrutinized role of NUhighepi, further substantiating our observations. Subsequently, we refined the cell-cell interaction analysis in space employing stlearn, a method that capitalizes on spatial information and gene expression profiles to pinpoint locations in the tissue where high ligand-receptor interaction activity. Cell-Cell Interaction (CCI) Analysis unveiled that COL1A1/COL1A2_ITGB1 constituted the more potent LR of the interaction between fibroblast and NME1 + epi/NME1-epi (Supplementary Fig. 9A-B). The outcome signified that spatial CCI of tumor cells was correlated with fibroblasts, which have been demonstrated to secrete deoxycytidine, a phenomenon that might induce compensatory tumor nucleoside salvage pathway flux under the constraint of de novo synthesis in vivo [35]. From the above results, it is evident that NME1 + Epi has higher nucleotide metabolic activity and a good dependency relationship with stromal cells (fibroblasts), similar to the biological behavior of NUhighepi.

**Fig. 7:**
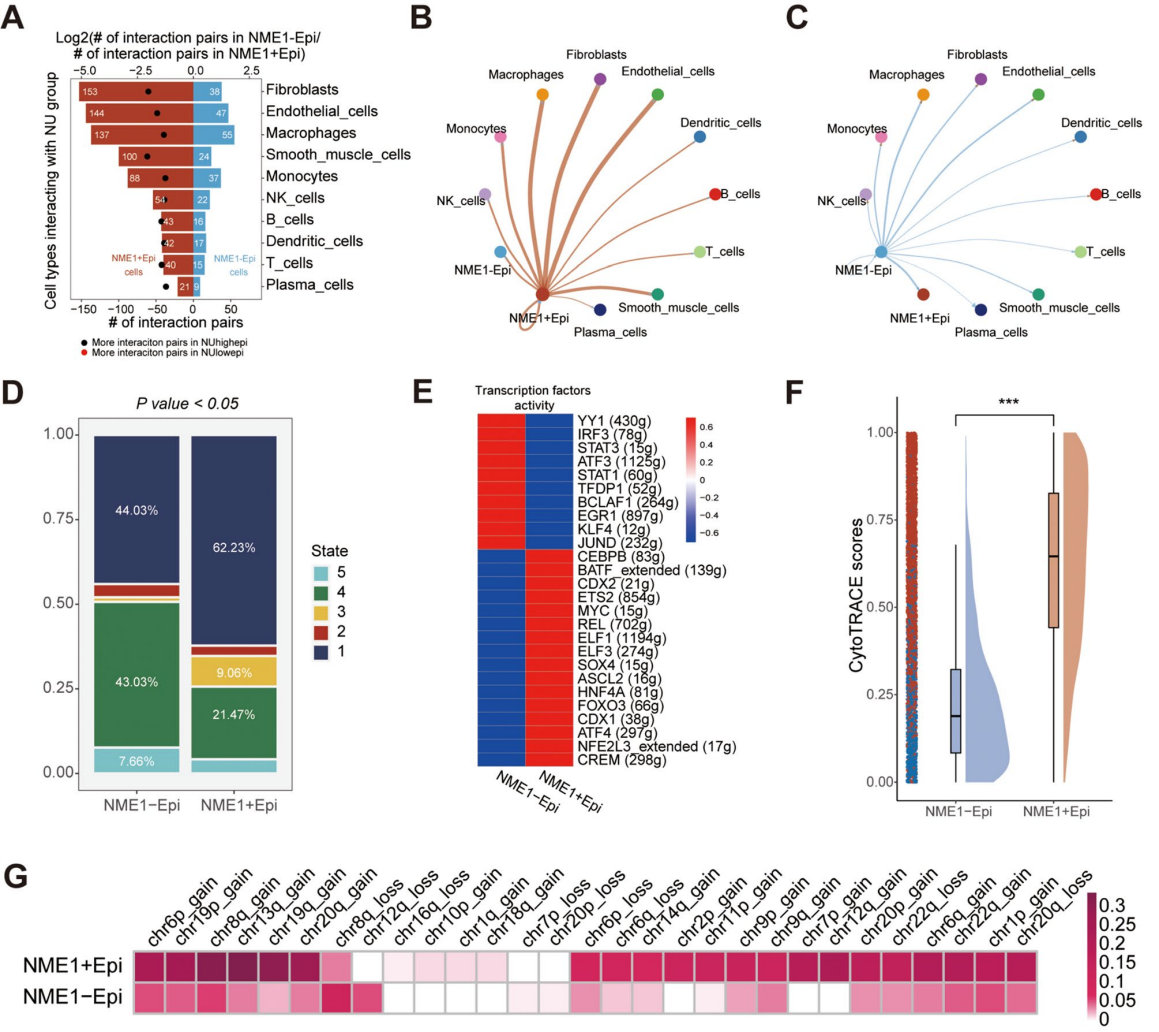
Single-cell transcriptome analysis of NME1 group: (**A**) Barplot showed the number of other cell types communicating with NME1 group. (**B**-**C**) Network plot visualizes the number of NME1 + epi/NME1-epi communicating with others. (**D**) Barplot showed pseudotime states of NME1 group. (**E**) Heatmap showing transcriptional activity of NU group. Intensity of color indicates average transcriptional activity. (**F**) Raincloud plot of CytoTRACE scores by NME1 group in scRNA-seq data. The center of the box plot are median values, the bounds of the box are 25% and 75% quantiles. (**G**) Heatmap showed inferCNV profiles of NME1 group. The P value of two-sided log-rank test is shown. ****P < 0.0001, ***P < 0.001, **P < 0.01, *P < 0.05, ns *P* > 0.05

We conducted an analysis of the interactions between epithelial cells (NME1 + epi and NME1-epi) and other clustered cells utilizing Cellphoedb in conjunction with scRNA-seq data. It was discerned that the quantity of potential ligand-receptor pairs between NME1 + epi and other cellular units is markedly elevated in comparison to NME1-epi (Fig. 7A). The cellular constituents that demonstrate an augmented interaction with NME + epi encompass fibroblasts, endothelial cells, and macrophages, a finding that corroborates the previously examined NUhighepi observations. In contrast, the degree of interaction between NME-epi and other cellular constituents was found to be inconspicuous (Fig. 7B-C). A pseudotime analysis revealed that NME + epi harbored a higher proportion of state 1, indicative of an early stage (Fig. 7D). Moreover, cell-type specific regulators linked to immune responses, progression, or metastasis, including MYC, SOX4, BATF, ELF1, ELF3, and FOXO3, were distinctly enriched in NME + epi, as determined by the Regulon Specificity Score (Fig. 7E). The attribute of cancer stemness, as appraised by CytoTRACE, manifested a significant elevation in NME + epi relative to NME-epi (Fig. 7F).

Subsequently, we inquired into how epithelial cell subclusters evolved during malignant progression. Initially, it was ascertained that the copy number variance scores of NME + epi were notably elevated among chr1p_gain, chr2p_gain, chr7p_gain, chr8q_gain, chr12q_gain, chr13q_gain, chr19q_gain, chr20q_gain, and chr22q_gain (Fig. 7F), a result consistent with the formerly analyzed NUhighepi findings.

In light of the foregoing results, we discerned that NME1 can serve as an indicator of nucleotide metabolism, and NME1 + epi is indissolubly associated with cells within the tumor microenvironment. Its functional evaluation is significantly correlated with tumor stemness and metastasis. These conclusions are congruent with those pertaining to NUhighepi previously described.

### NME1 suppress migration, and stemness in CRC cells

**Fig. 8:**
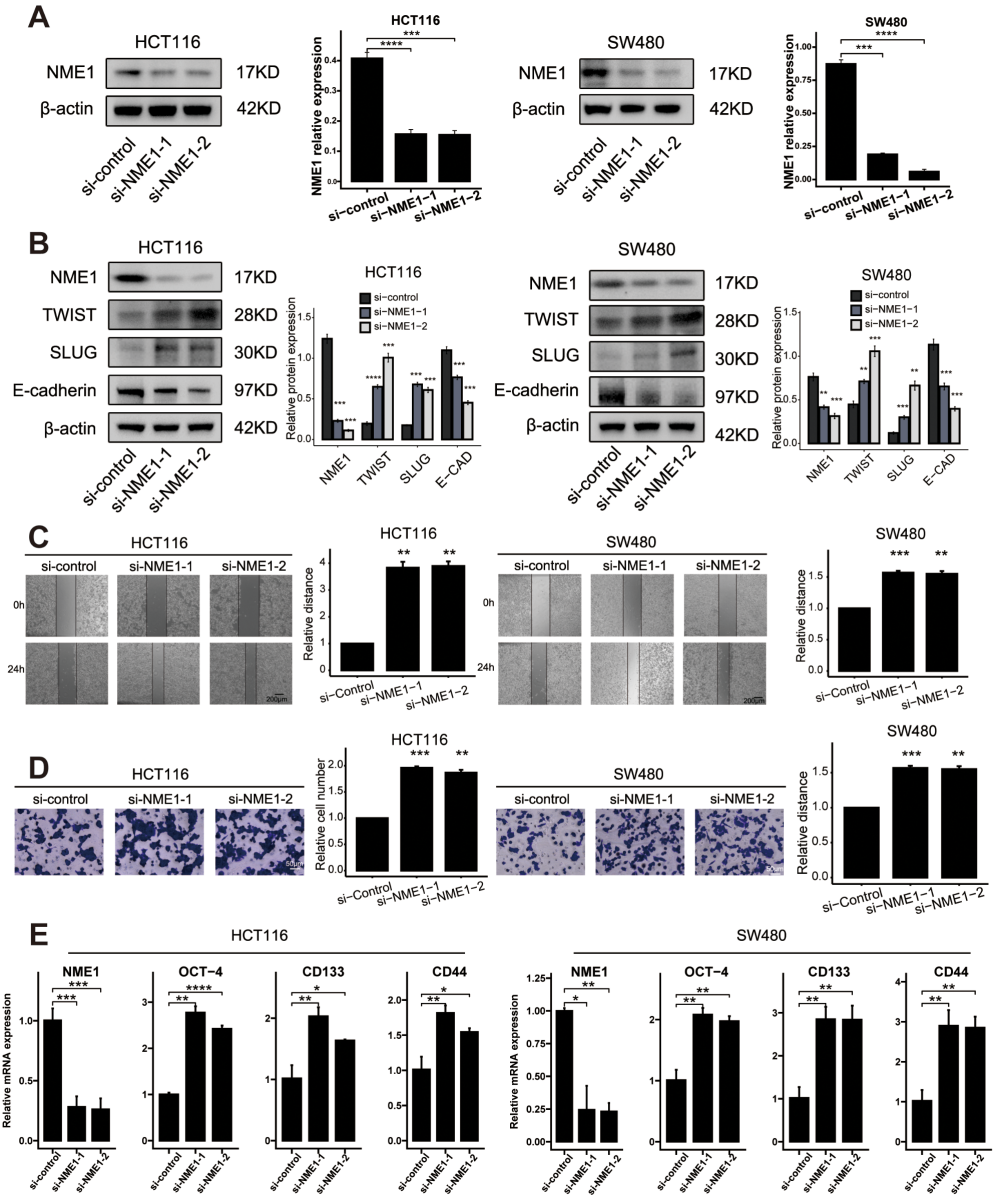
NME1 restrain tumor migration, and stemness in CRC cells.: (**A**) The level of NME1 transfection with siRNA were analyzed by WB. (**B**) WB analysis of the expression of EMT markers in CRC cells transfected with si-NME1. (**C**) Wound healing assays of cell migration in SW480 and HCT116 cells. The images of wound closureare presented at the indicated number of hours after scratching (0, 24 h). (**D**) Transwell assays were performed to examine the potential migration of si-NME1 cells or negative control cells. (**E**) RTq-PCR analysis of the expression of stemness markers in CRC cells transfected with si-NME1. ****P < 0.0001, ***P < 0.001, **P < 0.01, *P < 0.05, ns *P* > 0.05

In an effort to investigate the function of NME1, we suppressed the expression of these genes in HCT116 and SW480 cells through targeted siRNA. Two siRNAs specifically aimed at the coding region of NME1 were examined for their efficacy in knockdown (Fig. 8A). Western Blot (WB) analysis revealed that NME1 knockdown led to a diminution in the expression of TWIST and SLUG, and an augmentation in the expression of E-cadherin (Fig. 8B). Through an evaluation of preceding transcription factors, we ascertained that factors associated with transfer were conspicuously enriched. The WB analysis further corroborated that the silencing of NME1 could significantly inhibit the expression of TWIST and SLUG. Subsequent wound healing assays demonstrated that the silencing of NME1 amplified the migratory capability of SW480/HCT116 cells (Fig. 8C). These findings substantiate that NME1 may exert control over metastasis in colon cancer.

In harmony with these findings, the Transwell assay authenticated that the silencing of NME1 enhanced the invasiveness of HCT116 and SW480 cells (Fig. 8D). We then explored the effects of NME1 silencing on the downregulation of OCT-4, CD133, and CD44 at the mRNA levels (Fig. 8E). These observations propose that NME1 may have a prominent influence on the preservation of stemness within CRC cells.

### Construction of the NME1 risk score (NRS) by integrated machine learning

**Fig. 9:**
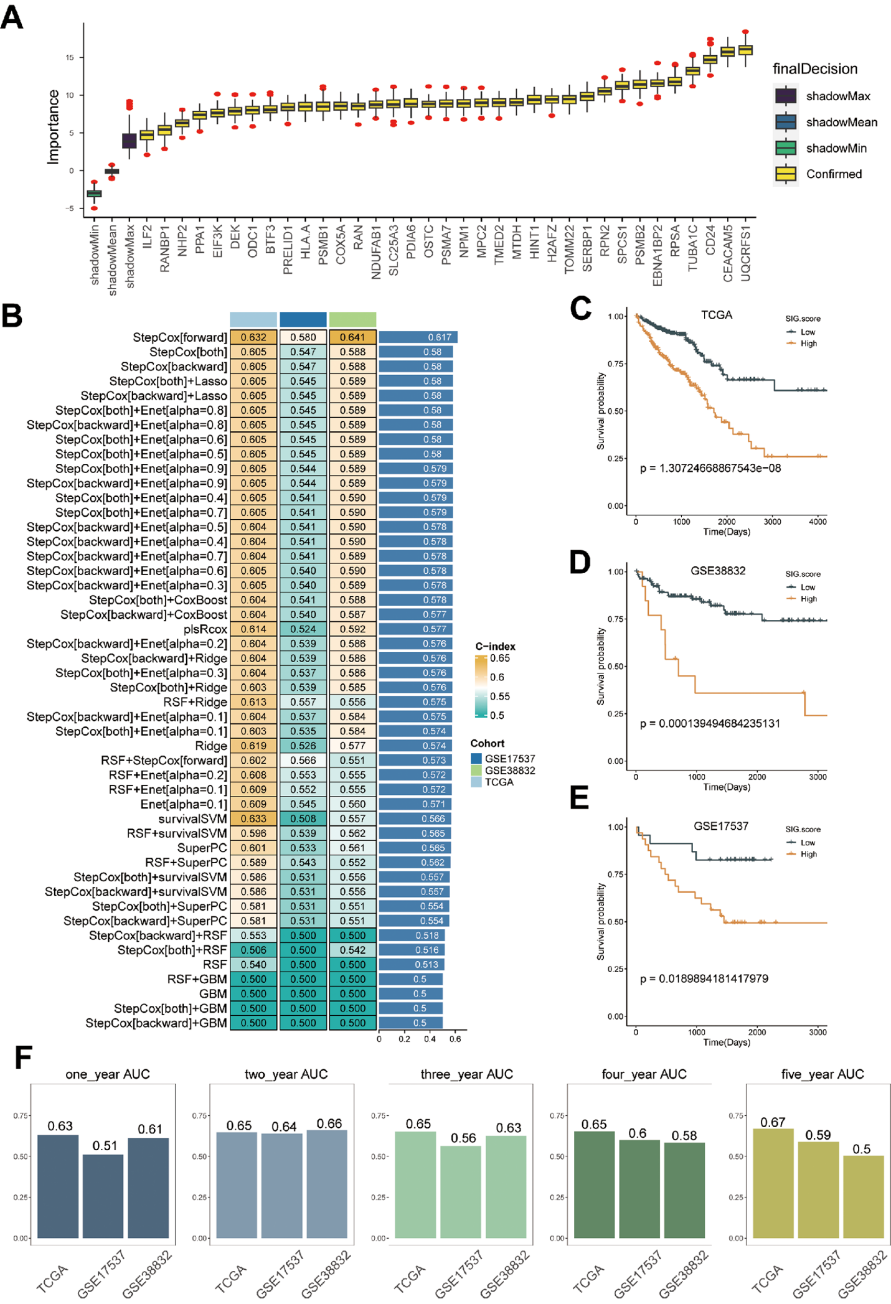
Machine learning-based gene signatures. (**A**) The Boruta algorithm identihed 47 NME1 + Epi related genes. Yellow epresents confirmed features while other colors denote shadow attributes. The corresponding boxplots compared the concordance index (C-index) values. (**B**) Machine learning was used to build 48 different models, and their C-indices were tested in each verification set. (**C**-**E**) Prognoses of patients in the TCGA (**C**), GSE17537 (**D**), and GSE38832 (**E**) cohorts. (**F**) Predicting patient survival at 1, 2, 3, 4, and 5 years using the NRS

By employing the Findmarkers, we identified the marker genes of NME1 + epi utilizing scRNA-data, leading to the discovery of a grand total of 168 genes (Supplementary Table 7). Univariate Cox regression analysis (Supplementary Table 8) revealed 140 genes that exhibited a significant association with overall survival. Furthermore, we employed the Boruta algorithm and narrowed down the selected genes to a group of 35 genes (Fig. 9A, Supplementary Table 9).

Next, we applied our machine learning algorithm to scrutinize these 35 genes and develop a predictive model (NME1 Risk Score, NRS). Utilizing 48 forecasting models, we examined the TCGA dataset and calculated the concordance index (C-index) for two validation datasets. Ultimately, the amalgamation of the StepCox algorithms yielded the most effective prototype, achieving an average C-index of 0.617 across all datasets (Fig. 9B). The TCGA training dataset and the two validation datasets showed that the high-risk group had significantly worse survival outcomes than the low-risk group (Fig. 9C-E). Based on the ROC analysis, NRS discriminated well with one-, two-, three-, four-, and five-year AUCs of 0.63,0.65,0.65,0.65, and 0.67 in the TCGA-CRC; 0.51,0.64,0.56,0.6,0.59 in GSE17537; 0.61,0.66,0.63,0.58,0.5 in GSE38832; These results indicate that the NRS performs effectively across various independent cohorts.

### The NRS has excellent predictive power for immunotherapy response

**Fig. 10:**
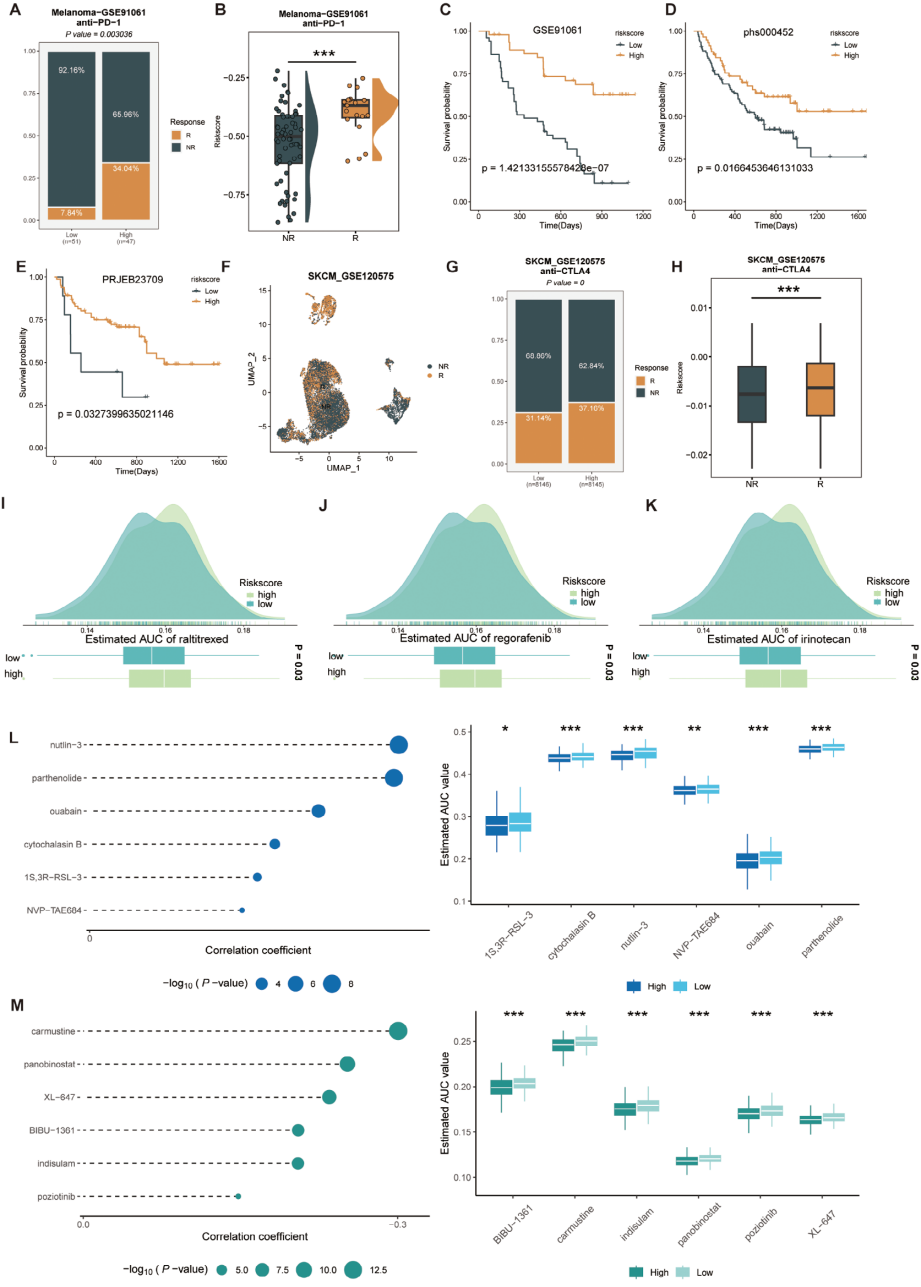
Predicting immune therapy response with NRS. (**A**) Comparison of the immune therapy response between groups with high and low NRS in GSE91061 cohort. (**B**) Comparison of the risk score between response/no response groups in GSE91061 cohort. (**C**-**E**) Based on the GSE91061 (**C**), phs000452 (**D**), and PRJEB23709 (**E**) immunotherapy cohorts, the effect of NRS on prognosis in these patients. (**F**) UMAP plot of single cells between response/no response groups in GSE120575 cohort. (**G**) Comparison of the immune therapy response between groups with high and low NRS in GSE120575 cohort. (**H**) Comparison of the risk score between response/no response groups in GSE120575 cohort. (**I**-**K**) Comparison of estimated raltitrexed’s (**I**), regorafenib’s (**J**), and irinotecan’s (**K**) sensitivity between Risk groups. (**L**-**M**) The correlation and differential analysis of drug sensitivity for potential drugs screened from the CTRP and PRISM datasets

To comprehensively assess the role of CMLS in MUC immunotherapy, we first investigated NRS and immune infiltration. The higher the risk score, the more infiltrated immune cells, such as T cells, B cells, macrophages, monocytes, endothelial cells, fibroblasts (Supplementary Fig. 10). However, there were no significant differences in the expression levels of each immune checkpoint among the risk groups. To further explore the differences in immunotherapy response between the high and low risk score groups, we constructed a risk score using public immunotherapy cohorts for differential comparison. The high-risk group had a higher percentage of patients who responded to immunotherapy, and the patients who responded to immunotherapy also had a higher risk score (Fig. 10A-B). The higher risk score group showed better prognostic outcomes and the same results were confirmed in three other immunotherapy cohorts, which indicates that the benefit of immunotherapy is greater (Fig. 10C-E). In the single-cell cohort, we found higher risk scores in cells that responded to immunotherapy, which is consistent with the previous results (Fig. 10F-H). In view of the poor response to immunotherapy in patients with low NRS, we comprehensively analyzed the CTRP and PRISM databases and found that the group with low risk scores was more sensitive to clinical drugs, such as: regorafenib, raltitrexed, and irinotecan (Fig. 10I-K). At the same time, we also screened drugs for high-risk scores, such as: nutlin-3, parthenolide, carmustine, and Panobinostat (Fig. 10L, M).

## Discussion

Nucleotide metabolism is perceived as the pivotal nexus in both tumorigenesis and the replication of cancer cells [36]. A plausible rationale for this phenomenon posits that the Tumor Microenvironment (TME) is incapable of furnishing adequate quantities or ratios of nucleotides unless proliferating cells augment the integrated metabolism of nonessential amino acids, ribose, and one-carbon donors to synthesize these intricate molecules [37]. Studies have shown that glutamine metabolism plays a significant role in regulating nucleotide metabolism [38, 39]. Glutamine is a nitrogen donor required for de novo synthesis of purines and pyrimidines, making it crucial for net nucleotide production during cell proliferation [40]. Therefore, inhibitors targeting glutamine metabolism are considered potential effective drugs for cancer treatment. Current strategies targeting tumor glutamine metabolism include systemic glutamine depletion, glutamine consumption within the tumor microenvironment, glutamine uptake inhibitors, glutamine antimetabolites, and glutaminase inhibitors. Studies have shown that bacterial L-asparaginase, a standard component of treatment protocols for acute lymphoblastic leukemia (ALL), can deplete plasma glutamine [41]. Colorectal cancer (CRC) ranks among the most ubiquitous malignancies globally and is associated with significant mortality rates. Thus, the downregulation of nucleotide metabolism may manifest as an efficacious strategy to either eradicate cancer cells or enhance the effectiveness of cancer treatments. In the present investigation, we have amalgamated scRNA-seq, spatial transcriptomics (ST), bulk RNA-seq, and foundational experimental methodologies to elucidate the malignant characteristics of nucleotide metabolism, examining both at the single-cell resolution and spatial organization levels.

In this context, we delineated the cellular and spatial immune landscape of nucleotide metabolism within colon cancer, deploying scRNA-seq in conjunction with spatial transcriptomics. Our findings unveiled that nucleotide metabolism is intensified within tumor epithelial cells, as determined through multiple scoring method calculations. Moreover, tumor cells exhibiting more robust nucleotide metabolism demonstrated an augmented potential for tumor stemness and growth, a conclusion that resonates with prior research [5, 6]. Specifically, the state referred to as NUhighepi was observed to be in a terminally differentiated state and manifested a heightened propensity for metastasis. This study not only demystifies a hitherto unexplored diversity within nucleotide metabolism and the immune system but also furnishes a comprehensive resource that stands to contribute significantly to the broader cancer research community.

The Macrophage Migration Inhibitory Factor (MIF) was among the inaugural cytokines to be identified and has emerged as an instrumental element in the genesis and advancement of colonic adenocarcinoma [42]. Utilizing NUhighepi/NUlowepi as a receptor, the ensuing signaling pathways were found to be closely related to MIF, thereby intimating that nucleotide metabolism and MIF are interwoven and consistently contribute to the exacerbation of colorectal cancer. Existing research corroborates that the gain of the 13q region is synonymous with a less favorable prognosis [43], fortifying our contention that nucleotide metabolism is a catalyst for the malignant evolution of colorectal cancer, a conclusion substantiated by the observed gain of the 13q region in NUhighepi. Through the employment of the scenic algorithm, we ascertained NUhighepi-specific transcription factors associated with tumor metastasis and progression, while the Cytotrace algorithm discerned more pronounced tumor stemness attributes within NUhighepi. Previous investigations have posited that aberrant nucleotide metabolism enables cancer cells to accentuate proliferation and progression [44]. In our quest to further substantiate the correlation between nucleotide metabolism and tumor progression and metastasis, we embarked on an exploration of the spatial transcriptome. Utilizing stlearn, an innovative Python package tailored for mapping the developmental trajectory predicated on tissue-wide SME normalized gene expression data, we astonishingly discerned that NUhighepi exhibited the apex of metastatic activity, insinuating that intratumor nucleotide metabolism of greater intensity correlates with an elevated metastatic propensity.

Emerging evidence accentuates that the altered manipulation of nucleotides by cancer cells paves the way for their immunological evasion via diverse mechanisms [45, 46]. Validation through the MISTy algorithm certified that NUhighepi frequently manifests colocalization or spatial adjacency with plasma cells, fibroblasts, and endothelial cells, predominantly through COL1A1/2_ITGB1 signaling pathways, as corroborated by the CCI network. It has become increasingly evident that Cancer-Associated Fibroblasts (CAFs) act as the principal wellspring of immunosuppressive activity within the TME [47]. Fibroblasts exert an influence on immune cell infiltration either directly—through the secretion of cytokines, chemokines, and cell surface proteins—or indirectly—via the deposition of various Extracellular Matrix (ECM) components and the remodeling of the matrix, upon which immune cells rely for intratumoral localization and migration. Ensuing research has illuminated that Tumor Endothelial Cells (TEC) contribute to the constitution of tumor immune tolerance under hypoxic conditions [48], and can engage with CAF through VEGFA [49]. In certain malignancies, TEC can amplify the immune checkpoint molecules of T cells, thereby inhibiting T cell activation [50], and TEC expressing FasL may mitigate the population of CD8 T cells while augmenting Treg numbers [51]. This implicates that these cells, concomitant with NUhighepi, may orchestrate immune subterfuge. Through meticulous spatial cell and pathway-dependent corroboration, we discerned that NUhighepi predominantly actuates Trail and Hypoxia, whereas NUlowepi chiefly activates estrogen. A contemporaneous study delineated how hypoxia-mediated acidification of the extracellular environment stymies the capacity of T cells to proliferate or enact cytotoxic effector functions [52]. In summation, nucleotide metabolism molds the tumor microenvironment by modulating cellular communication and the extent of reliance of tumor cells on specific pathways.

The essential role of NUhighepi in orchestrating tumor-promoting signaling pathways within the TME has led to the identification of core genes within your study. The resulting insights show a congruence between NME1 + epi and the malignant biological behavior of NUhighepi. In accordance with existing literature, NME1 has been recognized as a suppressor of metastasis, inhibiting tumor migration [53, 54]. This is resonant with our findings, which reveal higher survival rates among patients exhibiting high NME1 + epi, and further corroborated by evidence of suppressed migration and stemness within CRC cells. Consequently, our data coalesce to portray NME1 as an antagonist of tumor progression, wielding influence over the nucleotide metabolism of tumor cells and thus, indirectly sculpting the tumor microenvironment.

Studies have shown that nucleotide synthesis enzymes are highly druggable targets [55]. The types of drugs targeting nucleotide metabolism include dihydrofolate reductase inhibitors, thymidylate synthase inhibitors, dihydroorotate dehydrogenase inhibitors, inosine monophosphate dehydrogenase inhibitors, and ribonucleotide reductase inhibitors. 5-Fluorouracil (5-FU), methotrexate, and pemetrexed, as thymidylate synthase inhibitors, play important roles in the treatment of colorectal cancer. 5-FU is metabolized to 5-fluorodeoxyuridine monophosphate, which competitively inhibits thymidylate synthase by displacing its endogenous substrate, deoxyuridine monophosphate (dUMP) [56]. A drawback of this mechanism is that 5-FU induces the accumulation of dUMP, reducing its own effectiveness, as it must outcompete endogenous dUMP. Pemetrexed avoids this drawback by competitively inhibiting thymidylate synthase at the folate binding site, making its inhibitory activity unaffected by the accumulation of dUMP [57]. Although pemetrexed also inhibits DHFR and several enzymes in the purine de novo synthesis pathway, dTTP depletion is believed to be its primary cytotoxic mechanism [58]. Despite their exact mechanisms not being fully understood, methotrexate, 5-FU, and pemetrexed have demonstrated clinical efficacy and have become mainstays in the treatment of various cancers. Additionally, our research has identified several potential drugs targeting NME1, such as nutlin-3, parthenolide, ouabain, carmustine, and panobinostat, providing insights for further exploration of targeting nucleotide metabolism.

Despite these promising findings, the study does acknowledge certain limitations, most notably the restricted sample size. The focus of the investigation was concentrated on the biological attributes of NME1 + epi in cancer, a lens narrowed further by the exclusive study of a singular tumor type. Consequently, questions arise regarding the generalizability of these findings across diverse cancer manifestations. The mystery surrounding whether NME1 + epi represents a conserved subcluster common across various cancers further exacerbates this concern. The scarcity of direct evidence at the single-cell resolution of such epithelial cells also fuels the necessity for additional research.

Moving forward, a more expansive inquiry into the conserved characteristics of NME1 + epi across a spectrum of cancer types would be an invaluable step. This endeavor might include broadening the sample size and diversifying the tumor types under examination, applying integrative analyses that span both single-cell resolution and spatial dimensions, and possibly employing in vivo models to investigate the functional role of NME1 + epi within the broader context of tumor biology. Such efforts will not only contribute to a richer understanding of the functional role of NME1 in tumor progression but may also uncover novel therapeutic targets and prognostic markers for a variety of malignancies.

### Electronic supplementary material

Below is the link to the electronic supplementary material.

Supplementary Material 1: Nucleotide metabolism is increased in tumor epithelial cells: (A) Bubble plot visualizes the top 3 maker genes of each cellular population in GSE132257. (B) Cell number (upper) or ratio (lower) in diverse tissues inferred by scRNA-seq in GSE132257. (C) Uniform manifold approximation and projection (UMAP) plots of the colon cancer cells, colored by scoring, grouped by tissue types in GSE132257. ****P < 0.0001, ***P < 0.001, **P < 0.01, *P < 0.05, ns *P* > 0.05.

Supplementary Material 2: Nucleotide metabolism is increased in tumor epithelial cells: (A) UMAP plots of the colon cancer cells, colored by cell type in GSE132465. (B) Bubble plot of multi-methods of Nucleotide metabolism score in diverse celltype in GSE132465. (C) Violin plots of the scoring, faceted by tissue types in GSE132465. (D) UMAP plots of the colon cancer cells, colored by cell type in GSE144735. (E) Bubble plot of multi-methods of Nucleotide metabolism score in diverse celltype in GSE144735. (F) Violin plots of the scoring, faceted by tissue types in GSE144735. ****P < 0.0001, ***P < 0.001, **P < 0.01, *P < 0.05, ns *P* > 0.05.

Supplementary Material 3: Nucleotide metabolism is increased in tumor epithelial cells: (A) UMAP plots of the colon cancer cells, colored by cell type in GSE200997. (B) Bubble plot of multi-methods of Nucleotide metabolism score in diverse celltype in GSE200997. (C) Violin plots of the scoring, faceted by tissue types in GSE200997.

Supplementary Material 4: Communication between NU group and other cell types. (A) Barplot showed the number of other cell types communicating with NUhighepi/NUhighepi, considering the NU group as either receptor or ligand. (B-E) The heatmap showed that the receptor ligand pairs were between NU group and plasma cells (B), fibroblasts (C), endothelial cells (D), and macrophages (E). (F-H) Heatmap showed receptor ligand pairs of fibroblasts (F), endothelial cells (G), macrophages (H) communicating with NU group.

Supplementary Material 5: Copy number variation and differential expression analysis of NUhighepi/NUlowepi in scRNA-seq data. (A) Heatmap showed inferCNV profiles of NUhighepi and NUlowepi. (B) The violin plot showed the differential expression of nucleotide metabolism-related genes in normal and tumor.

Supplementary Material 6: Robust cell type decomposition (RCTD) annotation and pathway activity analysis in Spatial transcriptome. (A) RCTD annotation of NUgroup celltypes in Spatial transcriptome. (B) Pathway activity scores based on the PROGENy algorithm. Spatial slices showed the distribution of pathway activity, while violin plots illustrate the comparison of pathway activity between NUhighepi and NUlowepi.

Supplementary Material 7: The marker genes of cell type in scRNA data.

Supplementary Material 8: The primers sequences designated for RT-PCR.

Supplementary Material 9: The most significant interacting pair between NUhighepi/ NUlowepi and other cells.

Supplementary Material 10: Differential analysis of genes associated with nucleotide metabolism in normal and tumor cells.

Supplementary Material 11: The analysis of cell-cell interactions between NUhighepi/ NUlowepi and other cells within spatial contexts employing stlearn.

Supplementary Material 12: Gene Ontology (GO) enrichment analysis of Ligand-Receptors.

Supplementary Material 13: The marker genes of NME1 + epi in scRNA data.

Supplementary Material 14: Analysis of cell spatial dependency and pathway activity. (A) Cell-type abundances within a spot, and in the immediate or extended neighbourhood on the prediction of pathway activities inferred from spatially contextualized models. (B) Pathway activity scores based on the PROGENy algorithm. Spatial slices showed the distribution of pathway activity.

Supplementary Material 15: Inferring cancer-immune cell interactions by ligand-receptor (L-R) interactions and spatial trajectory analysis. (A) Analysis of ligand-receptor pair scoring and co-localization of ligand-receptor pairs. (B) Gene Ontology (GO) enrichment analysis of ligand-receptor (L-R) interactions. (C) A chord diagram illustrates the spatial communication between cells. (D-F) Spatial trajectory analysis-associated genes at ST-P1 (D), ST-P2 (E), ST-P4 (F), (red negative) (blue positive).

Supplementary Material 16: Cell-Cell Interaction (CCI) Analysis of NME1 group. (A) Strength of cell communication among all types of cells. (B) The communication strength of ligand-receptor pairs between cell types.

Supplementary Material 17: Immune function analysis of risk-score groups.

Supplementary Material 18: Univariate Cox regression analysis of the marker genes of NME1 + epi in scRNA data.

Supplementary Material 19: Boruta algorithm analysis of the marker genes of NME1 + epi in scRNA data.

## Data Availability

The open-access datasets are available through the following URL: GSE132257, GSE132465, GSE144735, GSE200997 (https://www.ncbi.nlm.nih.gov/geo), EMTAB8107 (https://www.ebi.ac.uk/biostudies/arrayexpress/studies/E-MTAB-8107), the ST-data (http://www.cancerdiversity.asia/scCRLM/), and the Cancer Genome Atlas (TCGA) database (https://xena.ucsc.edu). Genes correlated with nucleotide metabolism have been retrieved from the Gene Set Enrichment Analysis (GSEA) database (https://www.gsea-msigdb.org/gsea/index.jsp).
