# Supplementary figures and images for: Spatial transcriptome and single-cell reveal the role of nucleotide metabolism in colorectal cancer progression and tumor microenvironment
Source: J Transl Med. 2024 Jul 29;22:702. doi: 10.1186/s12967-024-05495-y (PMC11288102; doi:10.1186/s12967-024-05495-y)

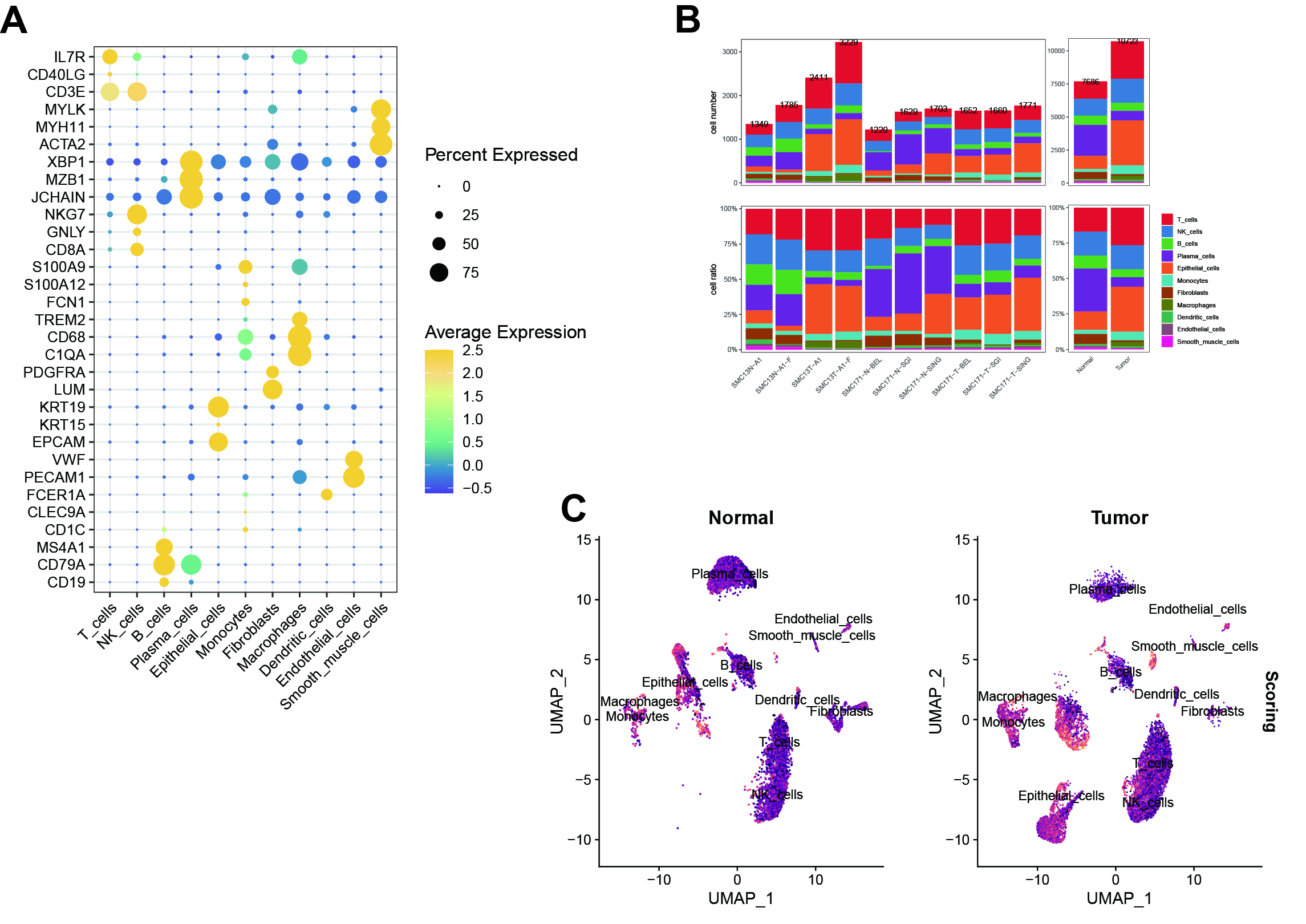

Supplement: Supplementary file 1 — Supplementary Material 1: Nucleotide metabolism is increased in tumor epithelial cells: (A) Bubble plot visualizes the top 3 maker genes of each cellular population in GSE132257. (B) Cell number (upper) or ratio (lower) in diverse tissues inferred by scRNA-seq in GSE132257. (C) Uniform manifold approximation and projection (UMAP) plots of the colon cancer cells, colored by scoring, grouped by tissue types in GSE132257. ****P < 0.0001, ***P < 0.001, **P < 0.01, *P < 0.05, ns P > 0.05. [file 12967_2024_5495_MOESM1_ESM.jpg]

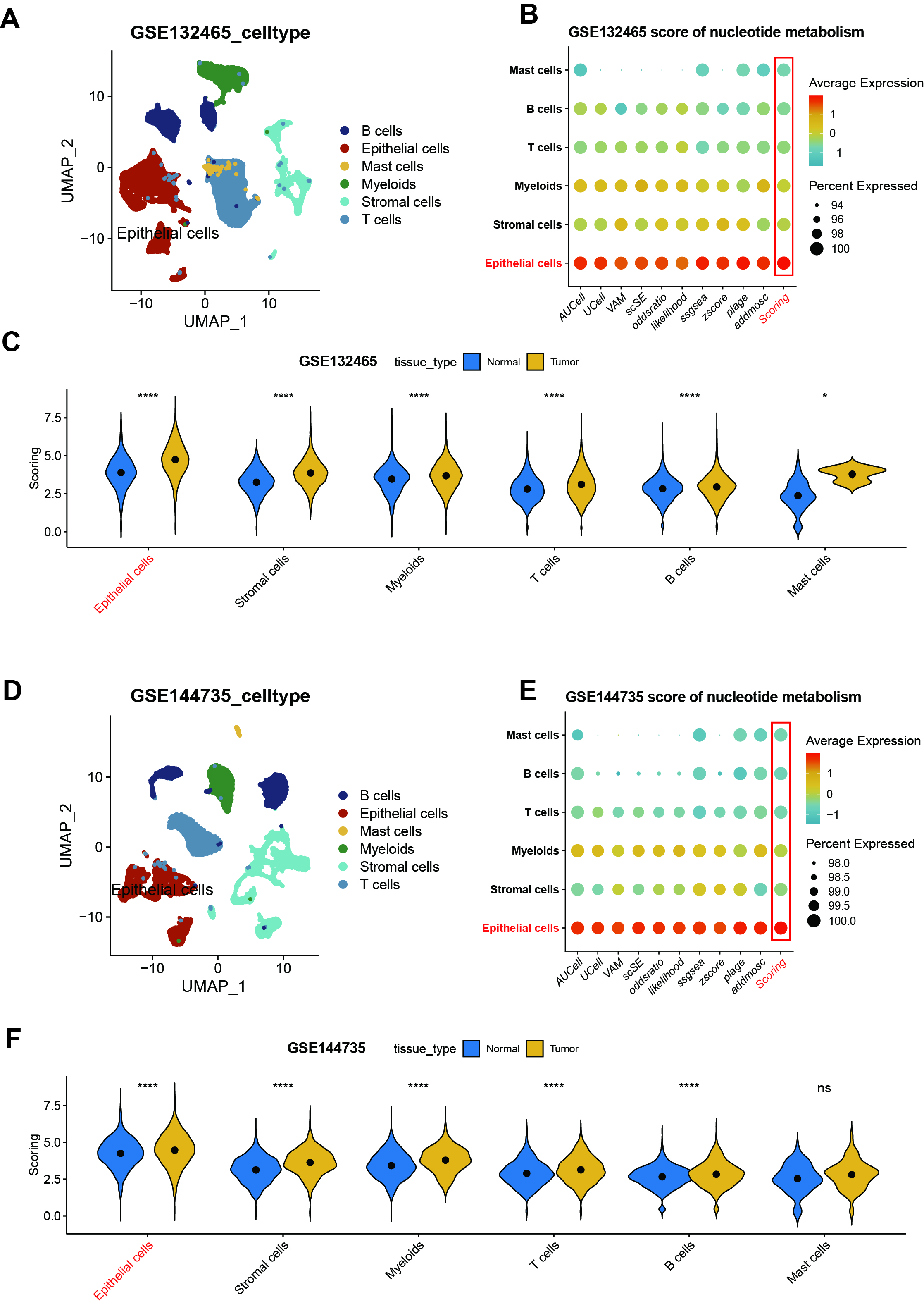

Supplement: Supplementary file 2 — Supplementary Material 2: Nucleotide metabolism is increased in tumor epithelial cells: (A) UMAP plots of the colon cancer cells, colored by cell type in GSE132465. (B) Bubble plot of multi-methods of Nucleotide metabolism score in diverse celltype in GSE132465. (C) Violin plots of the scoring, faceted by tissue types in GSE132465. (D) UMAP plots of the colon cancer cells, colored by cell type in GSE144735. (E) Bubble plot of multi-methods of Nucleotide metabolism score in diverse celltype in GSE144735. (F) Violin plots of the scoring, faceted by tissue types in GSE144735. ****P < 0.0001, ***P < 0.001, **P < 0.01, *P < 0.05, ns P > 0.05. [file 12967_2024_5495_MOESM2_ESM.jpg]

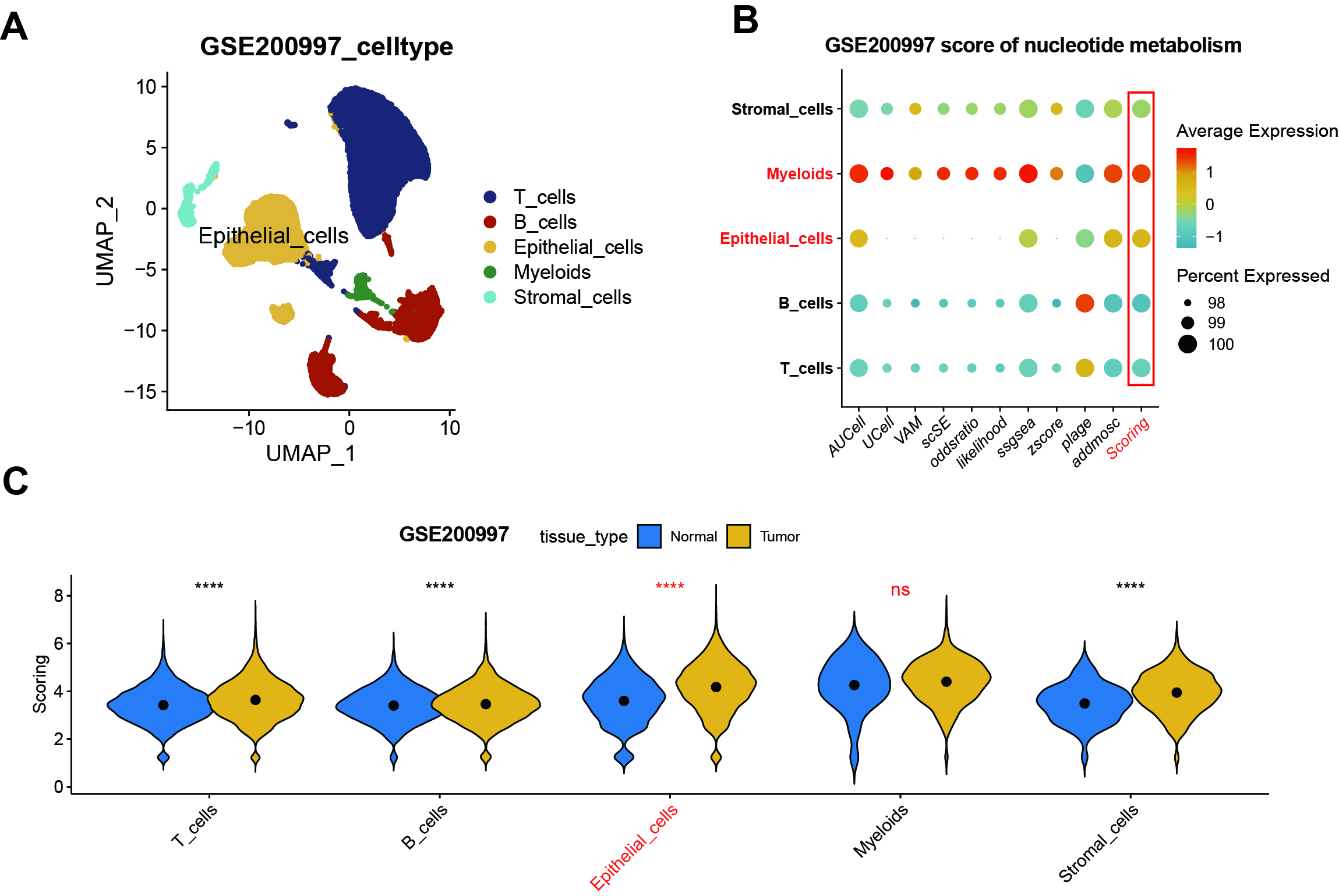

Supplement: Supplementary file 3 — Supplementary Material 3: Nucleotide metabolism is increased in tumor epithelial cells: (A) UMAP plots of the colon cancer cells, colored by cell type in GSE200997. (B) Bubble plot of multi-methods of Nucleotide metabolism score in diverse celltype in GSE200997. (C) Violin plots of the scoring, faceted by tissue types in GSE200997. [file 12967_2024_5495_MOESM3_ESM.jpg]

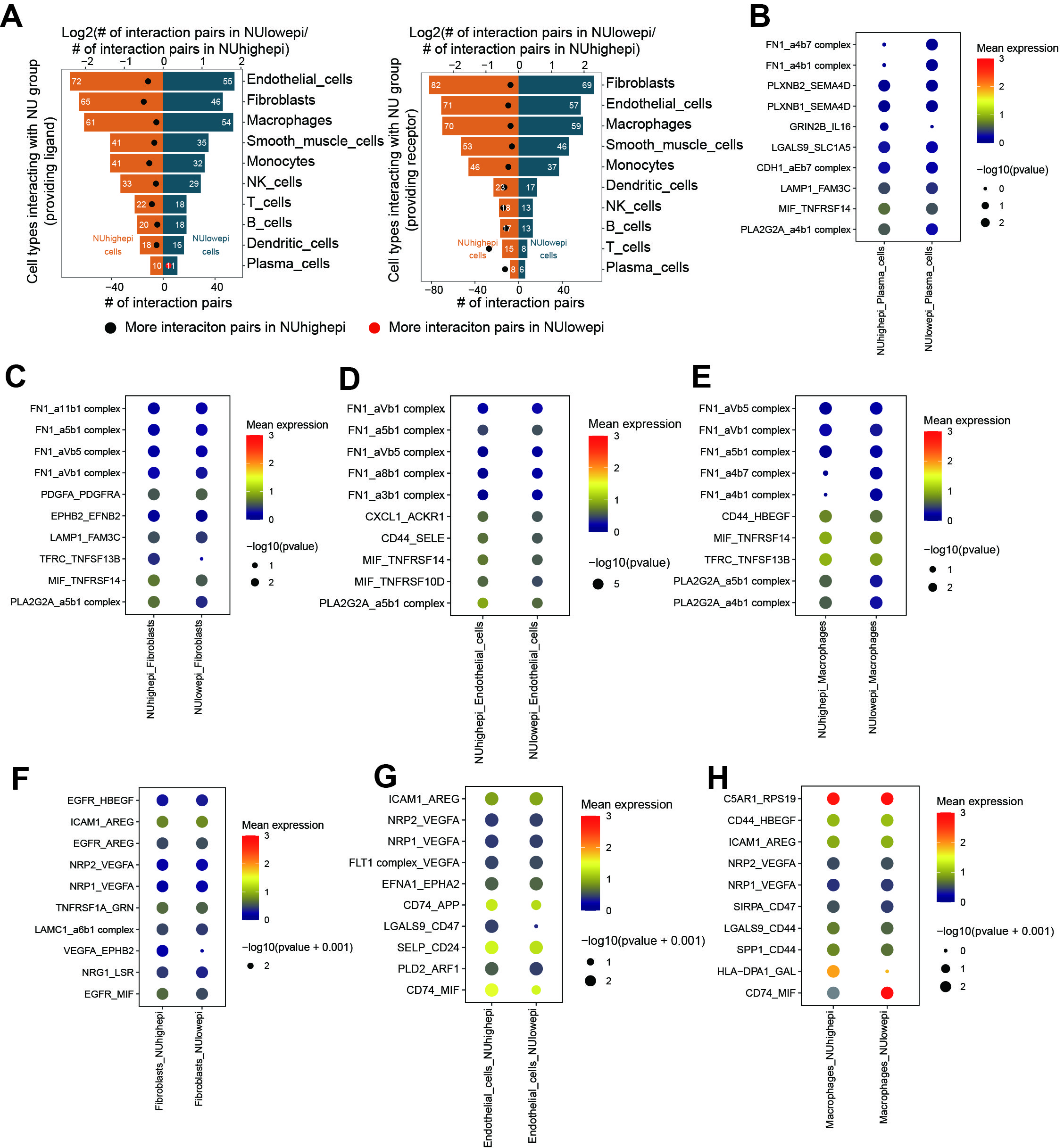

Supplement: Supplementary file 4 — Supplementary Material 4: Communication between NU group and other cell types. (A) Barplot showed the number of other cell types communicating with NUhighepi/NUhighepi, considering the NU group as either receptor or ligand. (B-E) The heatmap showed that the receptor ligand pairs were between NU group and plasma cells (B), fibroblasts (C), endothelial cells (D), and macrophages (E). (F-H) Heatmap showed receptor ligand pairs of fibroblasts (F), endothelial cells (G), macrophages (H) communicating with NU group. [file 12967_2024_5495_MOESM4_ESM.jpg]

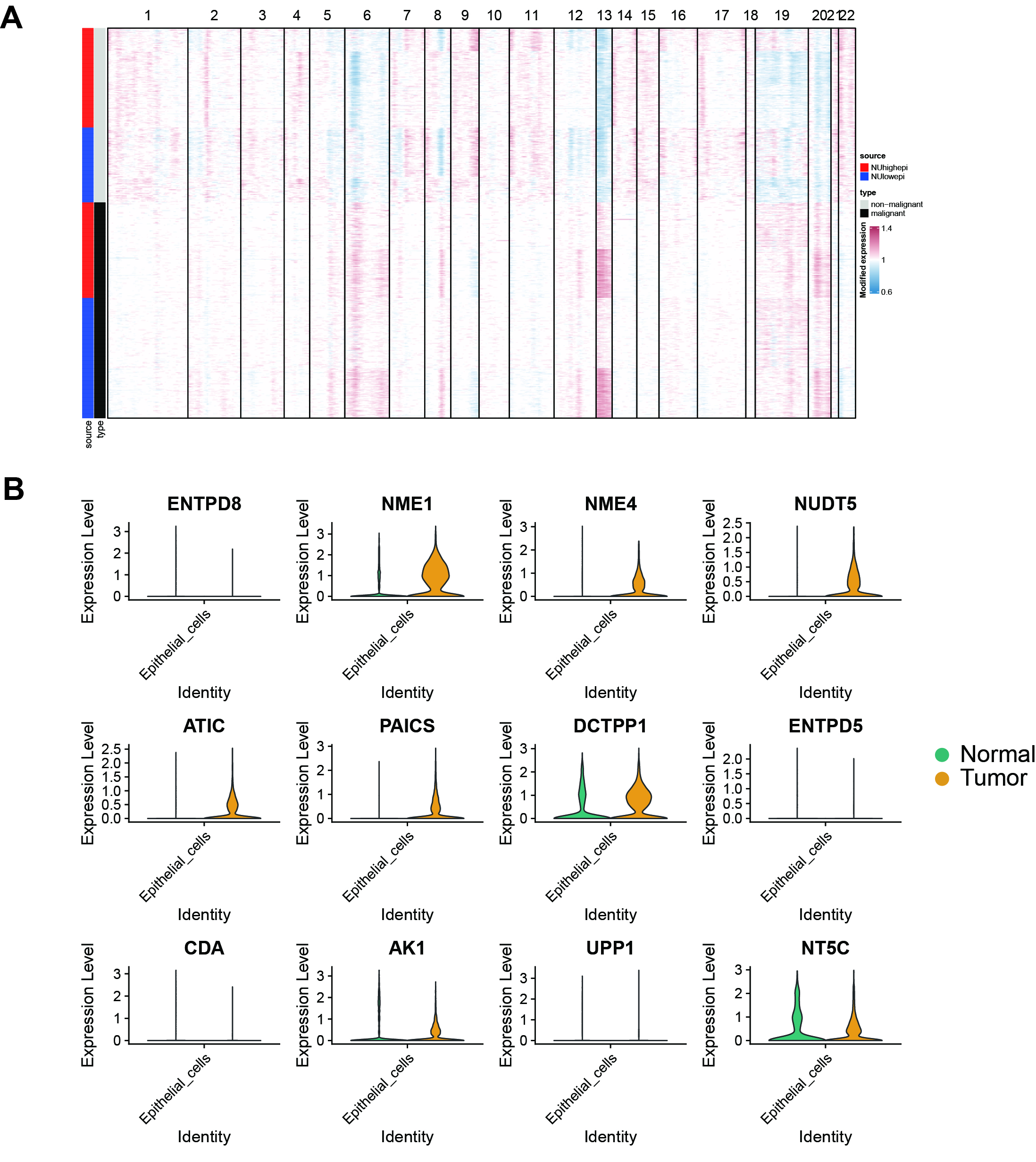

Supplement: Supplementary file 5 — Supplementary Material 5: Copy number variation and differential expression analysis of NUhighepi/NUlowepi in scRNA-seq data. (A) Heatmap showed inferCNV profiles of NUhighepi and NUlowepi. (B) The violin plot showed the differential expression of nucleotide metabolism-related genes in normal and tumor. [file 12967_2024_5495_MOESM5_ESM.jpg]

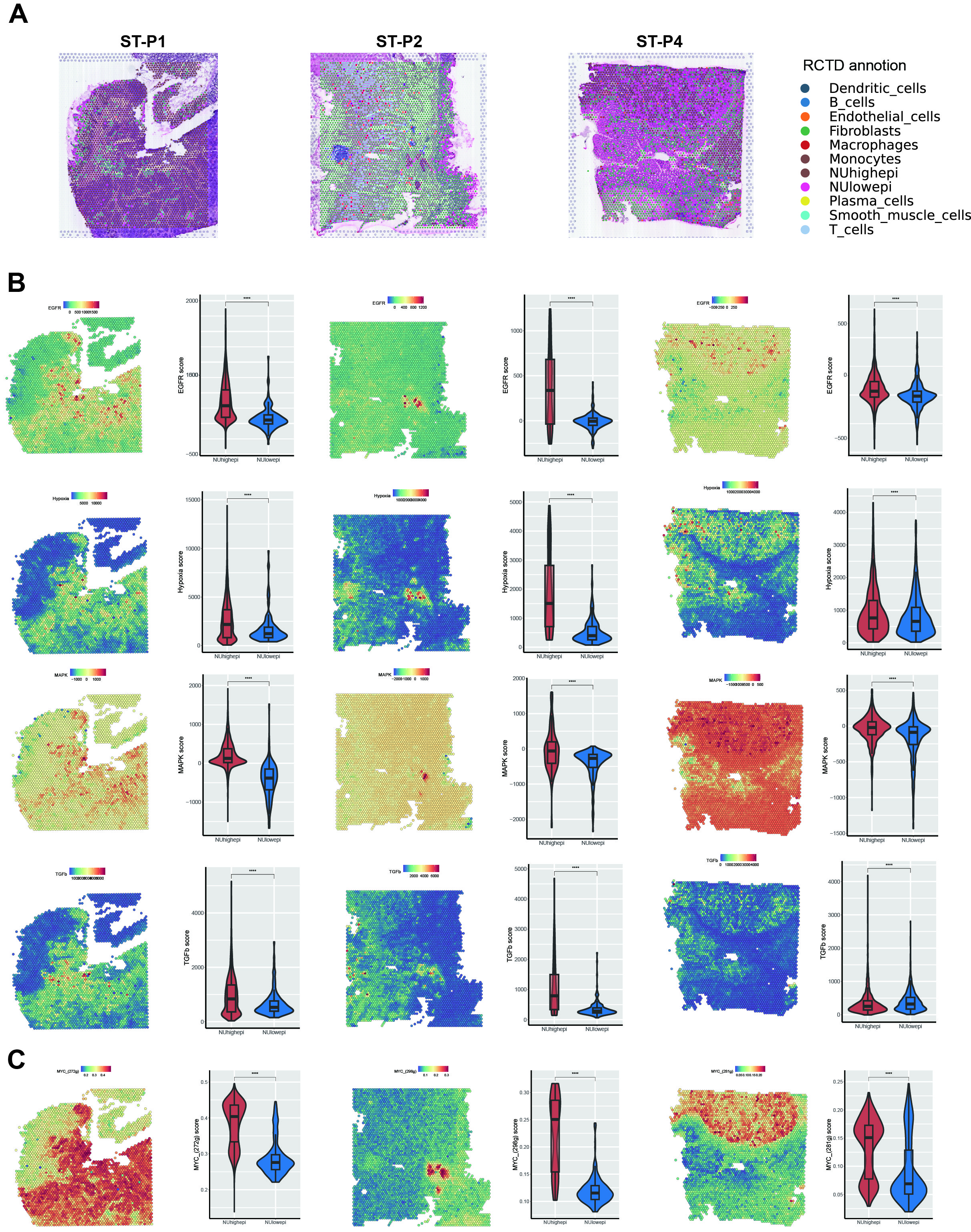

Supplement: Supplementary file 6 — Supplementary Material 6: Robust cell type decomposition (RCTD) annotation and pathway activity analysis in Spatial transcriptome. (A) RCTD annotation of NUgroup celltypes in Spatial transcriptome. (B) Pathway activity scores based on the PROGENy algorithm. Spatial slices showed the distribution of pathway activity, while violin plots illustrate the comparison of pathway activity between NUhighepi and NUlowepi. [file 12967_2024_5495_MOESM6_ESM.jpg]

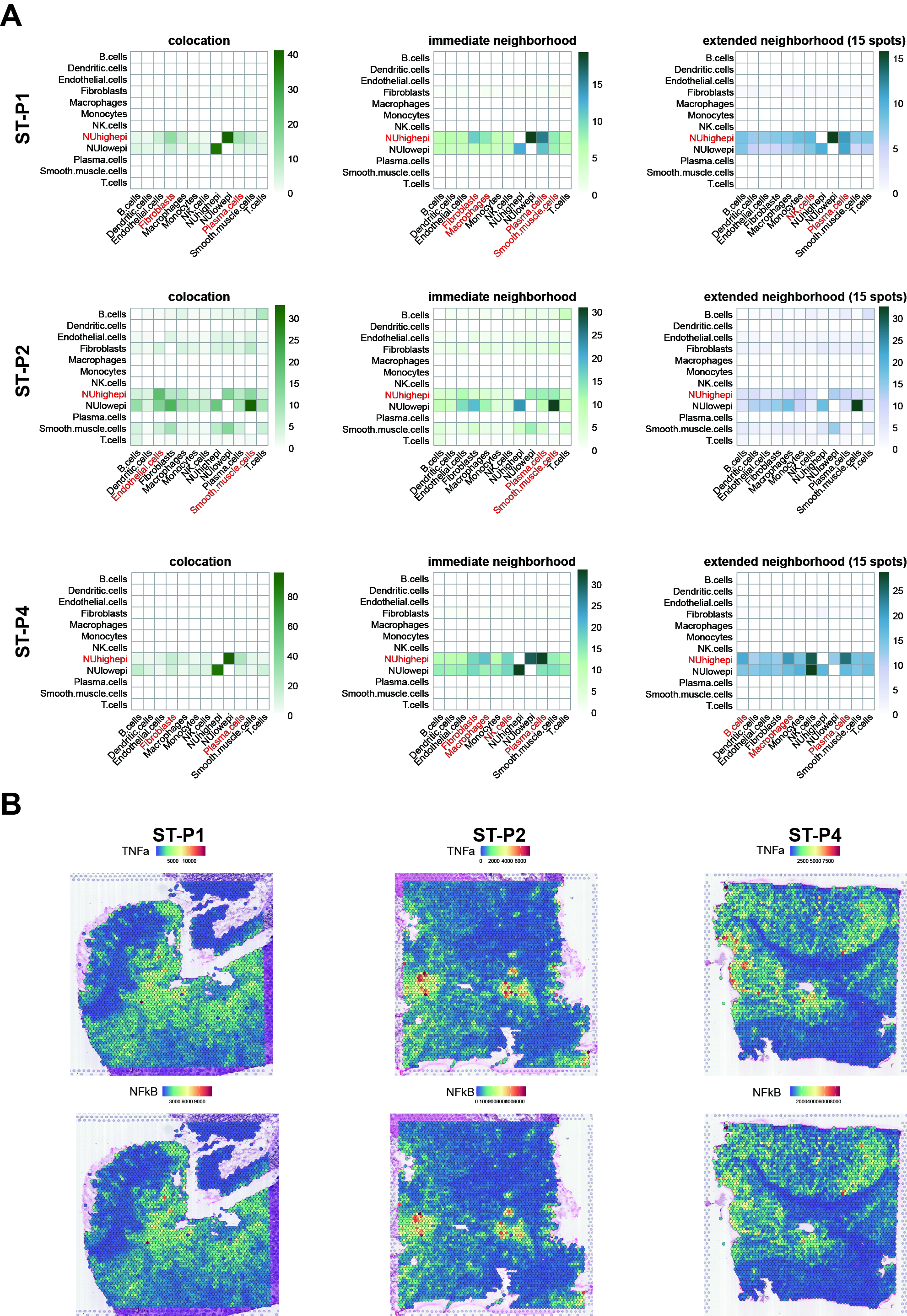

Supplement: Supplementary file 14 — Supplementary Material 14: Analysis of cell spatial dependency and pathway activity. (A) Cell-type abundances within a spot, and in the immediate or extended neighbourhood on the prediction of pathway activities inferred from spatially contextualized models. (B) Pathway activity scores based on the PROGENy algorithm. Spatial slices showed the distribution of pathway activity. [file 12967_2024_5495_MOESM14_ESM.jpg]

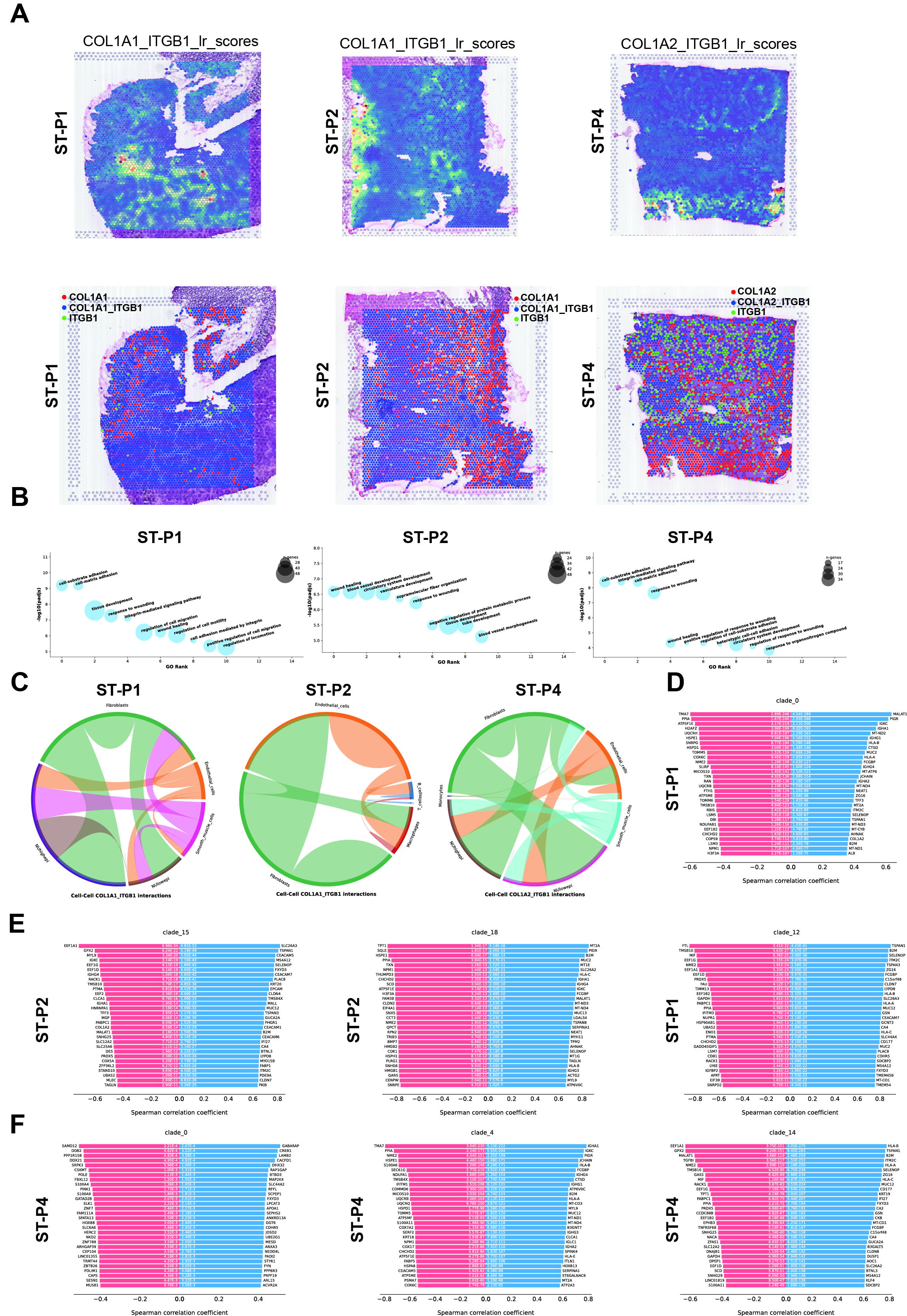

Supplement: Supplementary file 15 — Supplementary Material 15: Inferring cancer-immune cell interactions by ligand-receptor (L-R) interactions and spatial trajectory analysis. (A) Analysis of ligand-receptor pair scoring and co-localization of ligand-receptor pairs. (B) Gene Ontology (GO) enrichment analysis of ligand-receptor (L-R) interactions. (C) A chord diagram illustrates the spatial communication between cells. (D-F) Spatial trajectory analysis-associated genes at ST-P1 (D), ST-P2 (E), ST-P4 (F), (red negative) (blue positive). [file 12967_2024_5495_MOESM15_ESM.jpg]

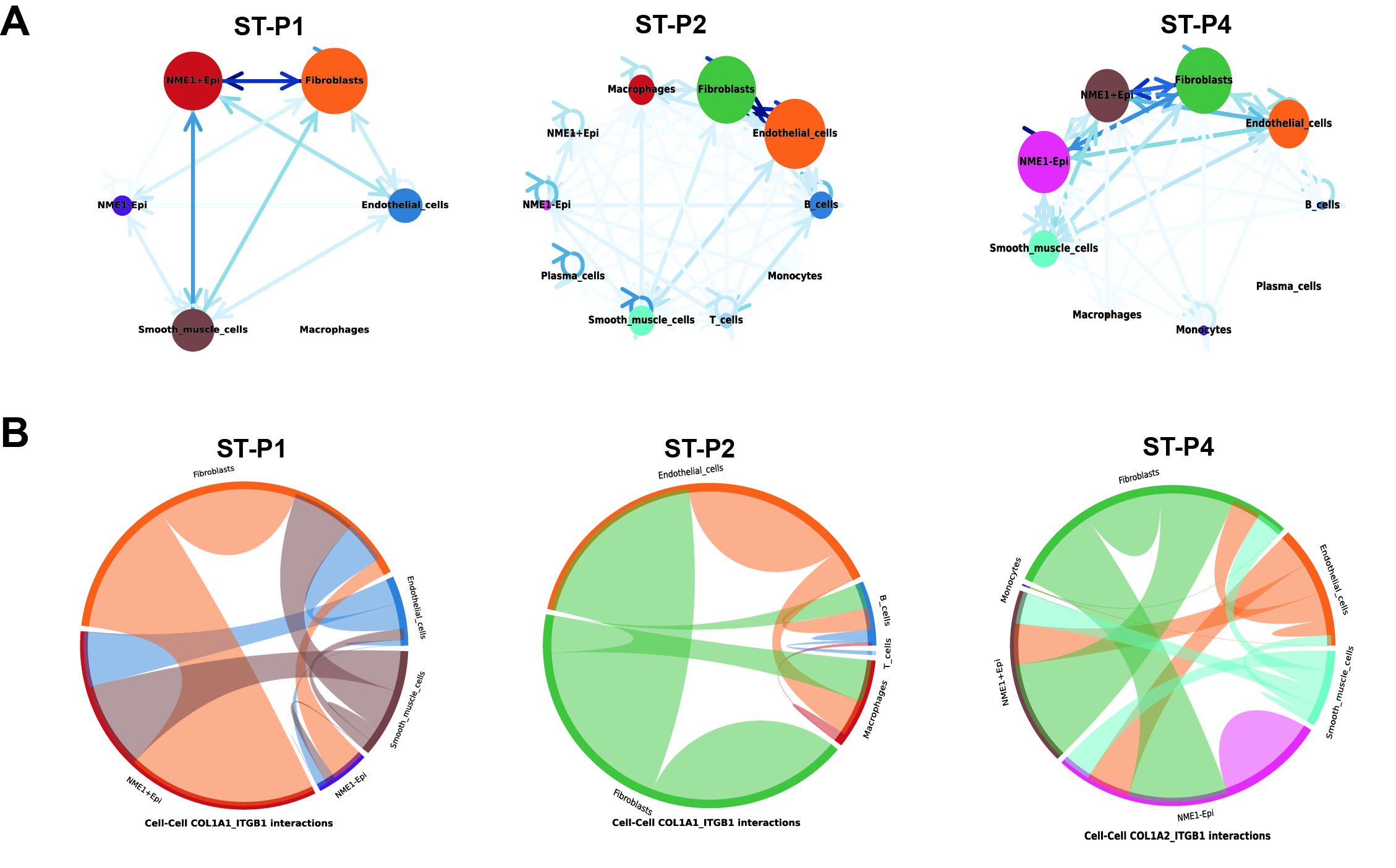

Supplement: Supplementary file 16 — Supplementary Material 16: Cell-Cell Interaction (CCI) Analysis of NME1 group. (A) Strength of cell communication among all types of cells. (B) The communication strength of ligand-receptor pairs between cell types. [file 12967_2024_5495_MOESM16_ESM.jpg]

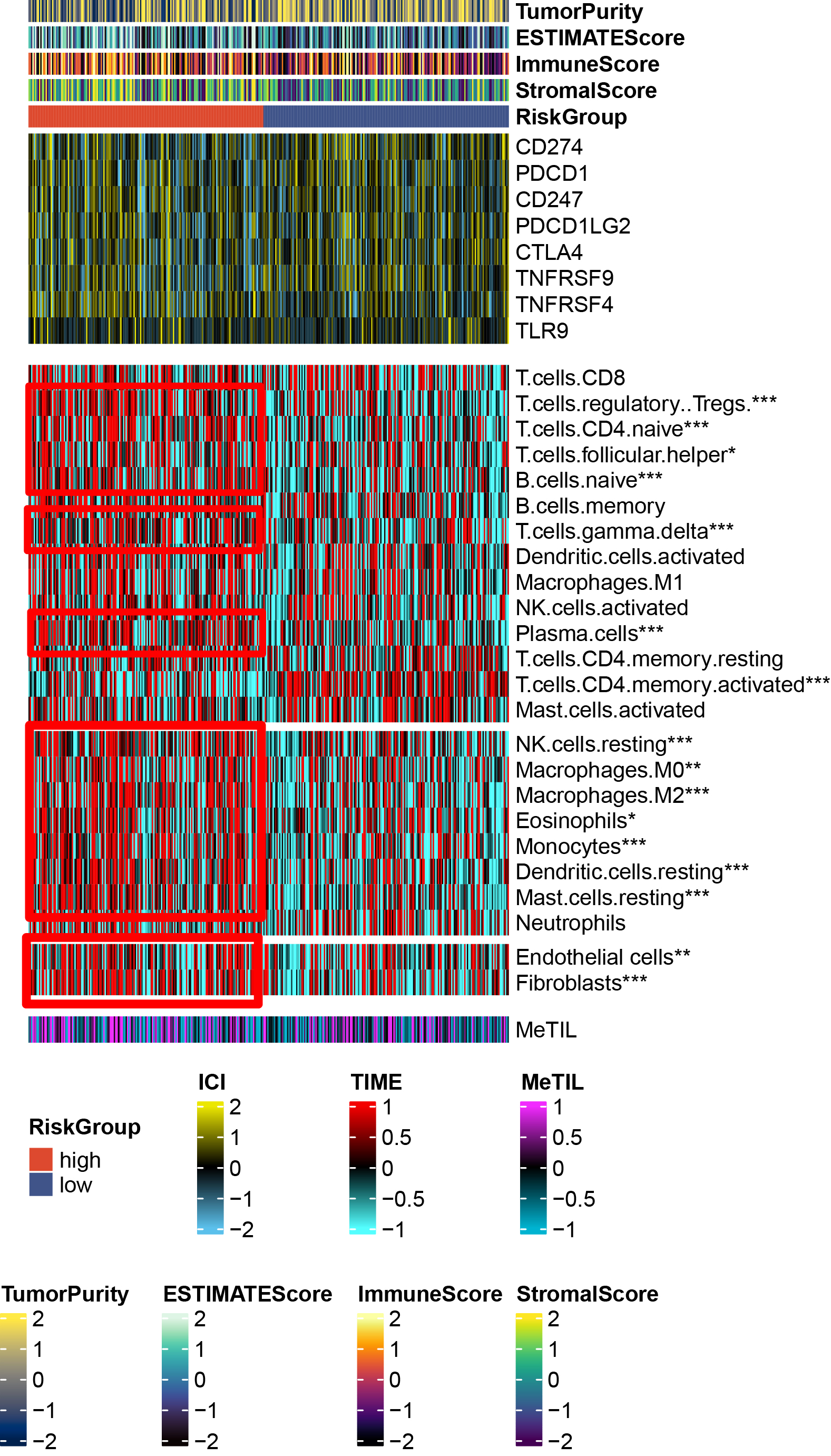

Supplement: Supplementary file 17 — Supplementary Material 17: Immune function analysis of risk-score groups. [file 12967_2024_5495_MOESM17_ESM.jpg]
